# Supplementary material for: Correction of Beta-Thalassemia IVS-II-654 Mutation in a Mouse Model Using Prime Editing
Source: Int J Mol Sci. 2022 May 25;23(11):5948. doi: 10.3390/ijms23115948 (PMC9180235; doi:10.3390/ijms23115948)
Supplement: Supplementary file 1 [file ijms-23-05948-s001.zip › ijms-1738368-supplementary.pdf]

Supplemental data

Correction of beta-thalassemia IVS-II-654 mutation in a mouse model using prime editing

Haokun Zhang [1], Ruilin Sun [2], Jian Fei [2], Hongyan Chen [1,*], and Daru Lu [1,3,*]

[1] State Key Laboratory of Genetic Engineering, MOE Engineering Research Center of Gene Technology, School of Life Sciences, Fudan University, Shanghai 200438, China; zhanghaokun666@hotmail.com (H.Z.)

[2] Shanghai Model Organisms Center, No.3577 Jinke Rd., Shanghai 201203, China; ruilin.sun@modelorg.com (R.S.); jian.fei@modelorg.com (J.F.)

[3] NHC Key Laboratory of Birth Defects and Reproductive Health, Chongqing Key Laboratory of Birth Defects and Reproductive Health, Chongqing Population and Family Planning, Science and Technology Research Institute, Chongqing 404100, China

* Correspondence: chenhy@fudan.edu.cn (H.C.); darulu@163.com (D.L.)

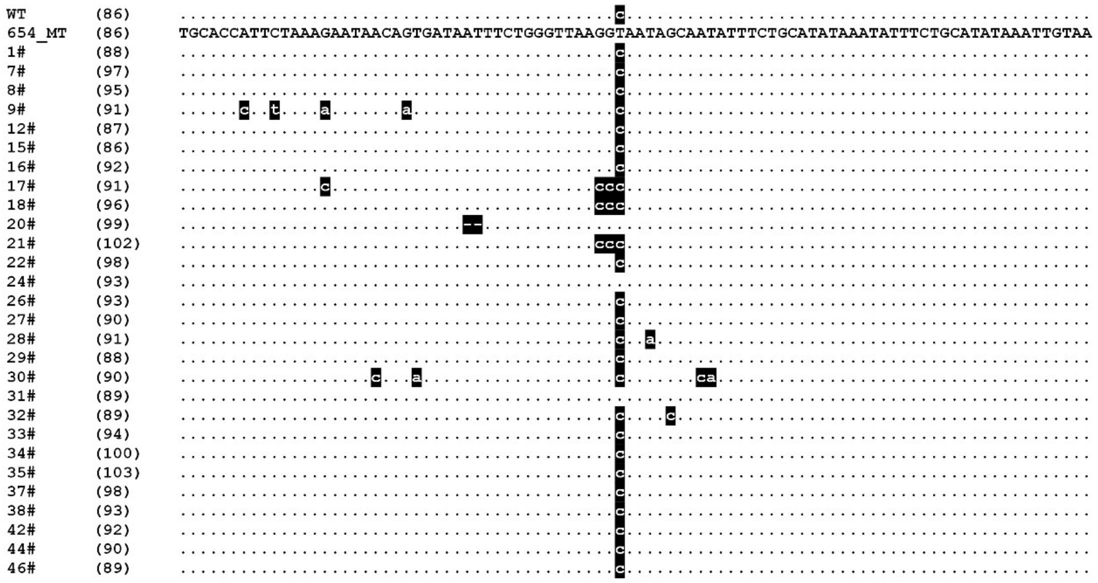

Figure S1. Alignment of Sanger sequencing at the IVS-II-654 site according to amplicons using genomic DNA from injected-zygote-derived mice. Alignment was performed to indicate differences from *Hbb^th-4^/Hbb^+^* (654_MT) mouse DNA sequence. Dots, dashes, and lowercase letters indicate identical nucleotides, deletions, and substitutions, respectively.

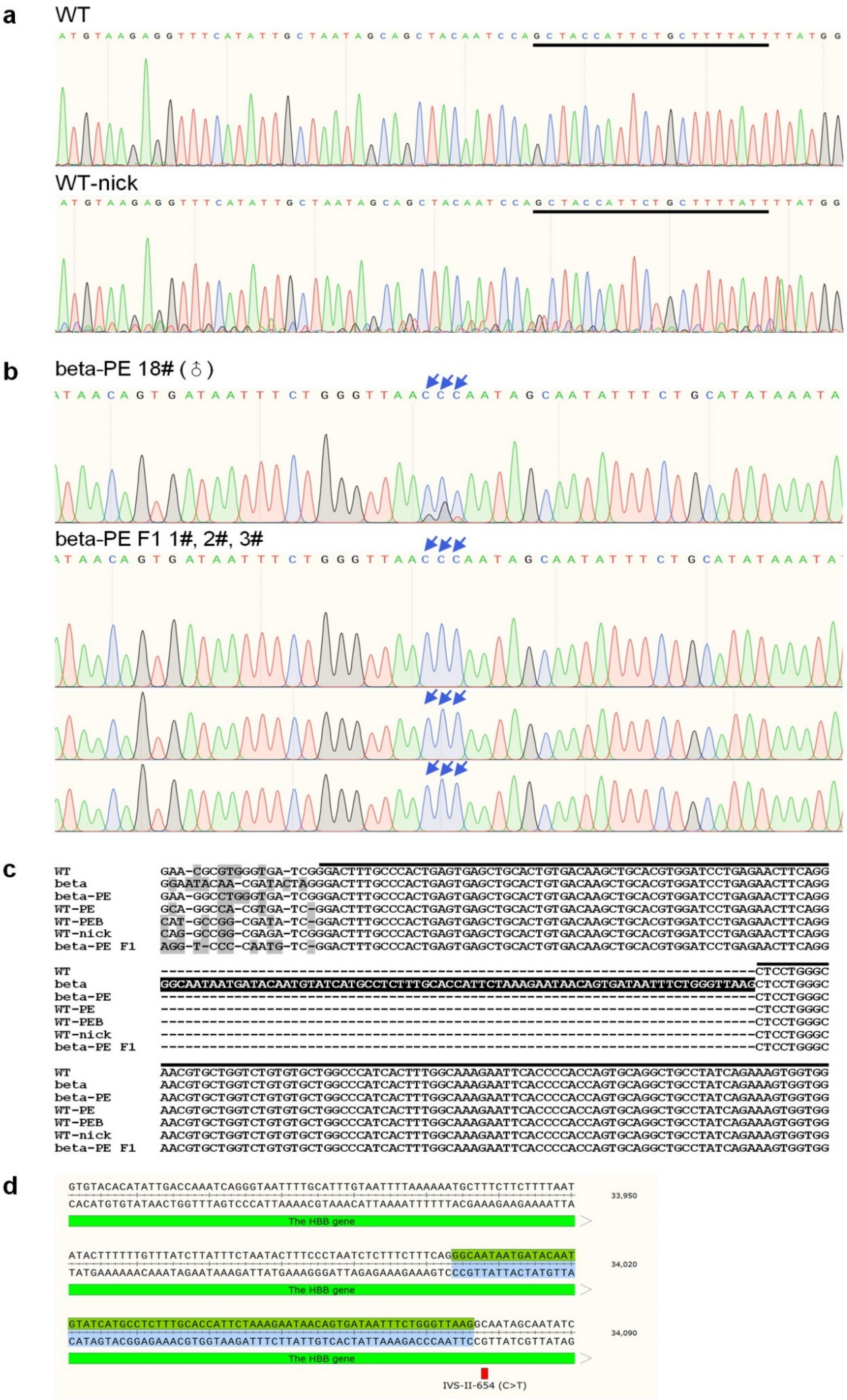

Figure S2 (related to Figure 2). (a) Sanger sequencing traces of WT and WT-

nick. Black line indicates genome position of Nick sgRNA. WT-nick trace shows continuous chaotic signals starting from the spacer sequence position of nick sgRNA. (b) Sanger sequencing traces at the IVS-II-654 site in beta-PE and beta-PE F1 pups. Traces show corrected editing (GGT>CCC) in beta-PE (18#) and F1 beta-genotype pups (1–3#). (c) Reverse transcriptase-polymerase chain reaction sequencing showed a 73-nucleotide (nt) sequence insertion in the *HBB* transcript in beta mice. (d) Alignment of the inserted 73-nt sequence. The inserted sequence in the *HBB* transcript in beta mice was consistent with the second intron sequence before the IVS-II-654 mutation site.

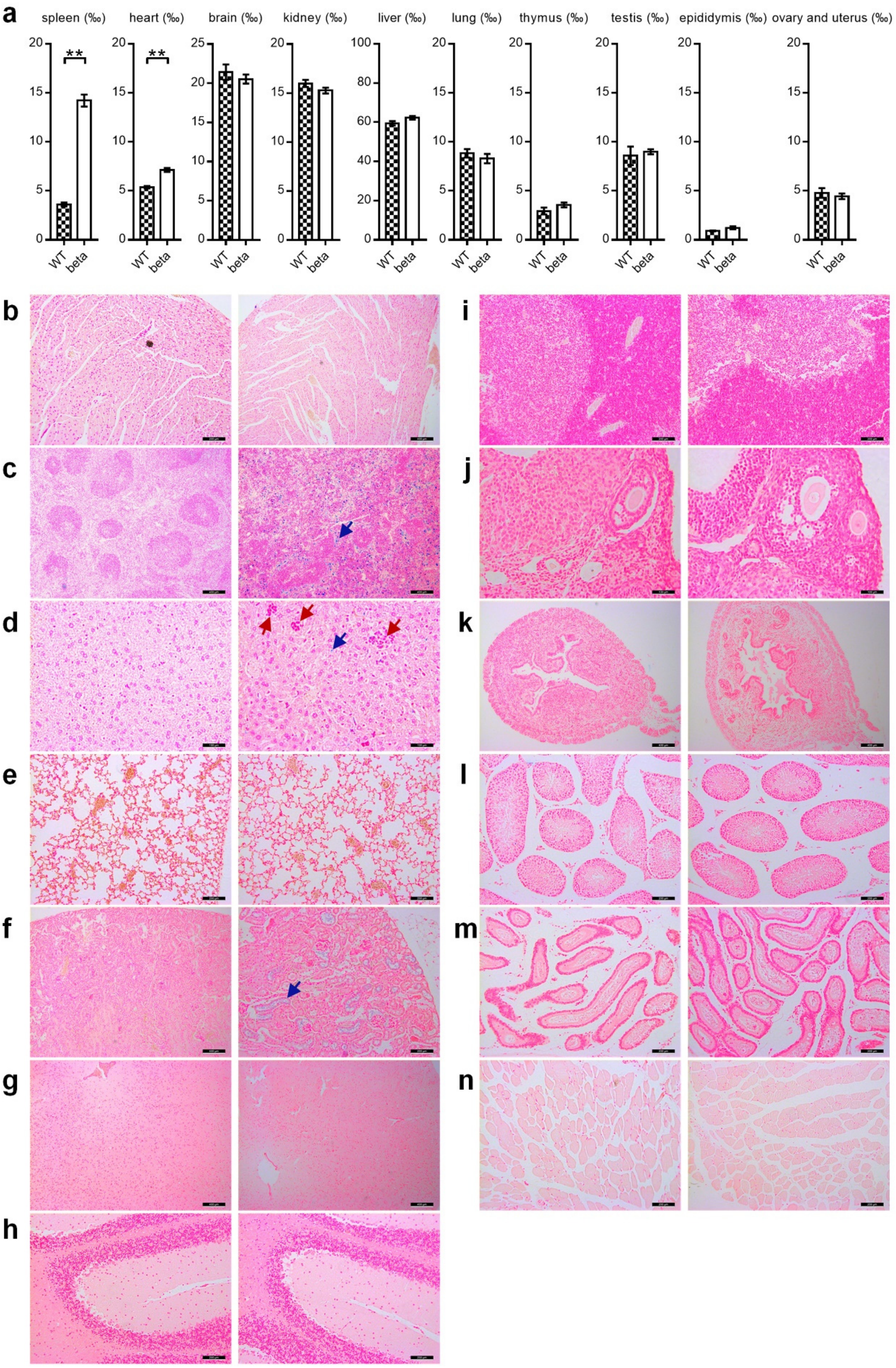

Figure S3 (related to Figure 3). (a) Tissue coefficient analysis between WT and beta mice. Values given as means ± standard error. $^{**}P < 0.01$ (t-test). Corresponding raw data are listed in Table S4. (b–n) Comparison of

ferrocyanide iron-stained tissue sections between WT and beta mice, including myocardium (b), spleen (c), liver (d), lung (e), kidney (f), forebrain (g), hindbrain (h), thymus (i), uterus (j), ovary (k), testis (l), epididymis (m), and muscle (c). Crimson and navy arrows indicate extramedullary hematopoiesis and iron deposition, respectively.

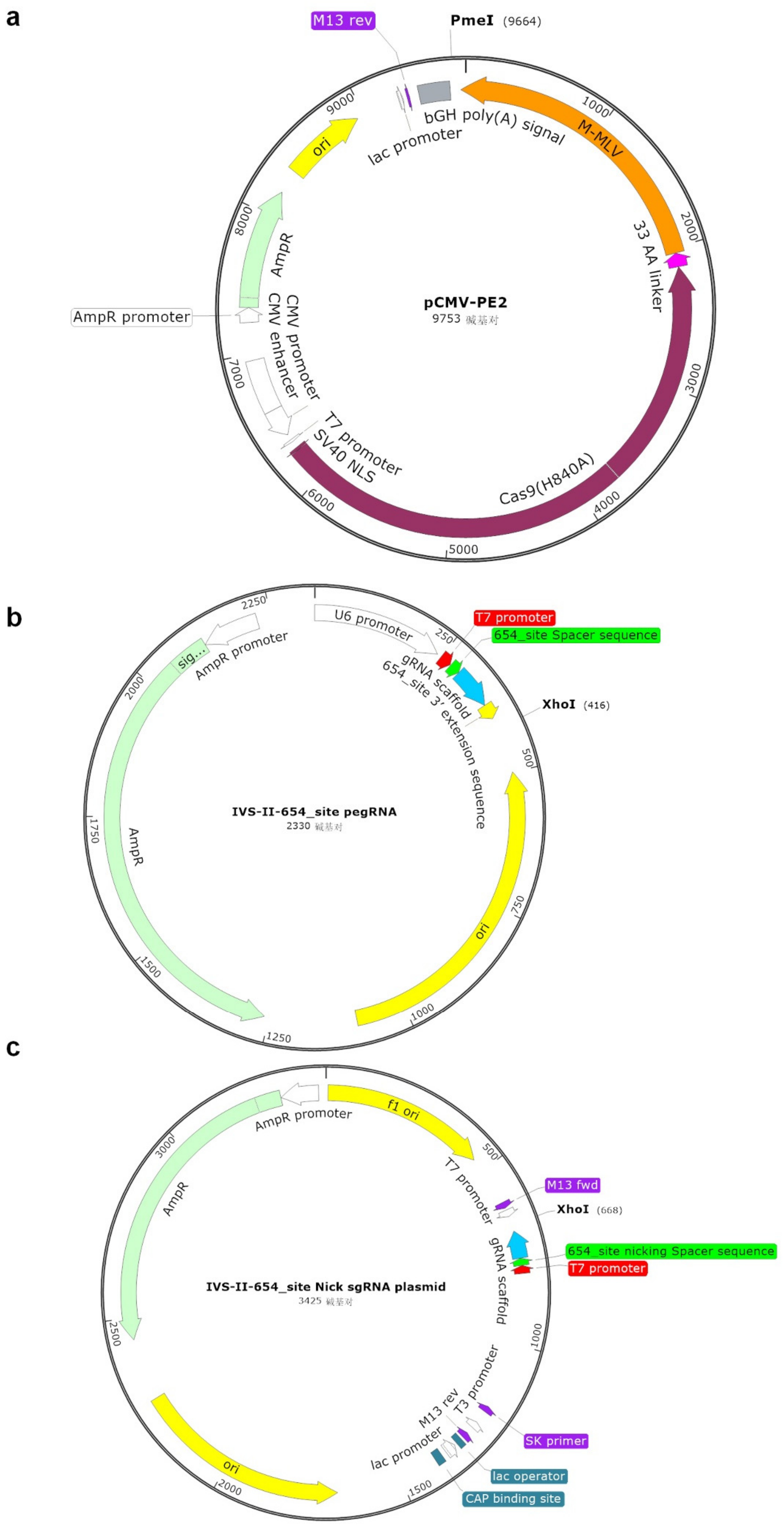
a
M13 rev
PmeI (9664)
bGH poly(A) signal
lac promoter
M-MLV
ori
AmpR
33 AA linker
pCMV-PE2
9753 碱基对
AmpR promoter
CMV promoter
CMV enhancer
T7 promoter
SV40 NLS
Cas9(H840A)
1000
2000
3000
4000
5000
6000
7000
8000
9000
b
2250
U6 promoter
250
T7 promoter
654_site Spacer sequence
sig...
AmpR promoter
gRNA scaffold
654_site 3' extension sequence
XhoI (416)
500
2000
1750
IVS-II-654_site pegRNA
2330 碱基对
AmpR
ori
750
1500
1000
1250
c
AmpR promoter
f1 ori
500
3000
T7 promoter
M13 fwd
XhoI (668)
AmpR
654_site nicking Spacer sequence
T7 promoter
gRNA scaffold
IVS-II-654_site Nick sgRNA plasmid
3425 碱基对
2500
1000
T3 promoter
M13 rev
SK primer
lac promoter
lac operator
CAP binding site
ori
2000
1500

Figure S4. Plasmids required for PE3-induced correction of IVS-II-654 mutation. (a) pCMV-pe2 plasmid. (b, c) Plasmids of IVS-II-654 pegRNA (b) and IVS-II-654 nick sgRNA (c). Restriction sites of PmeI and XhoI labeled for linearization.

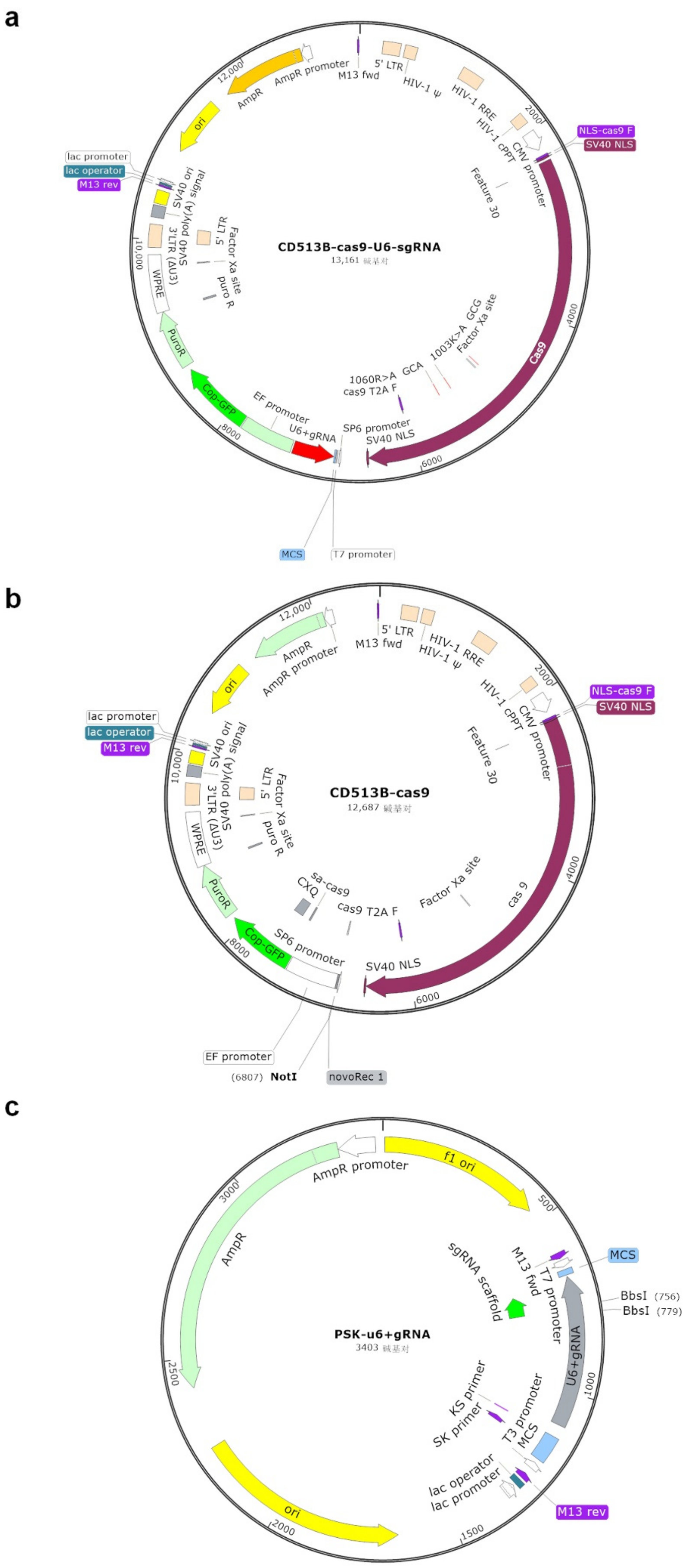
a
CD513B-cas9-U6-sgRNA
13,161
5' LTR
HIV-1 ψ
HIV-1 RRE
HIV-1 cPPT
CMV promoter
NLS-cas9 F
SV40 NLS
Feature 30
Cas9
1003K>A GCG
Factor Xa site
1060R>A GCA
cas9 T2A F
SP6 promoter
SV40 NLS
U6+gRNA
EF promoter
Cop-GFP
PuroR
WPRE
3'LTR (ΔU3)
SV40 poly(A) signal
SV40 ori
5' LTR
Factor Xa site
puro R
lac promoter
lac operator
M13 rev
ori
AmpR
AmpR promoter
M13 fwd
MCS
T7 promoter
2000
4000
6000
8000
10,000
12,000
b
CD513B-cas9
12,687
5' LTR
M13 fwd
HIV-1 ψ
HIV-1 RRE
HIV-1 cPPT
CMV promoter
NLS-cas9 F
SV40 NLS
Feature 30
cas 9
Factor Xa site
cas9 T2A F
sa-cas9
CXQ
SP6 promoter
SV40 NLS
Cop-GFP
PuroR
WPRE
3'LTR (ΔU3)
SV40 poly(A) signal
SV40 ori
5' LTR
Factor Xa site
puro R
lac promoter
lac operator
M13 rev
ori
AmpR
AmpR promoter
EF promoter
(6807) NotI
novoRec 1
2000
4000
6000
8000
10,000
12,000
c
PSK-u6+gRNA
3403
AmpR promoter
f1 ori
M13 fwd
T7 promoter
MCS
BbsI (756)
BbsI (779)
sgRNA scaffold
U6+gRNA
KS primer
SK primer
T3 promoter
MCS
lac operator
lac promoter
M13 rev
ori
AmpR
500
1000
1500
2000
2500
3000

Figure S5. Plasmids required for off-target analysis. (a) Modified plasmid containing Cas9, sgRNA, and the reporter gene *EGFP*. (b) The original CD513b-cas9 plasmid can be used to integrate the sgRNA into the NotI site (c) PSK-u6+gRNA plasmid. The BbsI site was used for sgRNA construction.

Figure S6

a

>PSK-u6+gRNA

CACCTGACGCGCCCTGTAGCGGCGCATTAAGCGCGGCGGGTGTGGTGGTTACGCGCAGCGTGACCGCTACACTTGCCAGCGCCCTAGCGCCCGCTCCTTTCGCTTTCTTCCCTTCCTTTCTCGCCACGTTCGC
CGGCTTTCCCCGTCAAGCTCTAAATCGGGGGCTCCCTTTAGGGTTCCGATTTAGTGCTTTACGGCACCTCGACCCCAAAAAACTTGATTAGGGTGATGGTTCACGTAGTGGGCCATCGCCCTGATAGACGGTT
TTTCGCCCTTTGACGTTGGAGTCCACGTTCTTTAATAGTGGACTCTTGTTCCAAACTGGAACAACACTCAACCCTATCTCGGTCTATTCTTTTGATTTATAAGGGATTTTGCCGATTTCGGCCTATTGGTTAA
AAAATGAGCTGATTTAACAAAAATTTAACGCGAATTTTAACAAAATATTAACGCTTACAATTTCCATTCGCCATTCAGGCTGCGCAACTGTTGGGAAGGGCGATCGGTGCGGGCCTCTTCGCTATTACGCCAG
CTGGCGAAAGGGGGATGTGCTGCAAGGCGATTAAGTTGGGTAACGCCAGGGTTTTCCCAGTCACGACGTTGTAAAACGACGGCCAGTGAATTGTAATACGACTCACTATAGGGCGAATTGGGTACCGGGCCCC
CCCTCGAGACTAGTAAAAAA**GCACCGACTCGGTGCCACTTTTTCAAGTTGATAACGGACTAGCCTTATTTTAACTTGCTATTTCTAGCTCTAAAAC**GTCTTCTGAGAAGACTGCAAACAAGGCTTTTCTCCA
AGGGATATTTATAGTCTCAAAACACACAATTACTTTACAGTTAGGGTGAGTTTCCTTTTGTGCTGTTTTTAAAATAATAATTTAGTATTTGTATCTCTTATAGAAATCCAAGCCTATCATGTAAAATGTAGC
TAGTATTAAAAAGAACAGATTATCTGTCTTTTATCGCACATTAAGCCTCTATAGTTACTAGGAAATATTATATGCAAATTAACCGGGGCAGGGGAGTAGCCGAGCTTCTCCCACAAGTCTGTGCGAGGGGGCC
GGCGCGGGCCTAGAGATGGCGCGTCGGATCTCTAGAGCGGCCCGGTACTAACCAAACTGGATATCGATAAGCTTGATATCGAATTCCTGCAGCCCGGGGGATCCACTAGTTCTAGAGCGGCCGCCACCGCGG
TGGAGCTCCAGCTTTTGTTCCCTTTAGTGAGGGTTAATTTCGAGCTTGGCGTAATCATGGTCATAGCTGTTTCCTGTGTGAAATTGTTATCCGCTCACAATTCCACACAACATACGAGCCGGAAGCATAAAGT
GTAAAGCCTGGGGTGCCTAATGAGTGAGCTAACTCACATTAATTGCGTTGCGCTCACTGCCCGCTTTCCAGTCGGGAAACCTGTCGTGCCAGCTGCATTAATGAATCGGCCAACGCGCGGGGAGAGGCGGTTT
GCGTATTGGGCGCTCTTCCGCTTCCTCGCTCACTGACTCGCTGCGCTCGGTCGTTCGGCTGCGGCGAGCGGTATCAGCTCACTCAAAGGCGGTAATACGGTTATCCACAGAATCAGGGGATAACGCAGGAAAG
AACATGTGAGCAAAAGGCCAGCAAAAGGCCAGGAACCGTAAAAAGGCCGCGTTGCTGGCGTTTTTCCATAGGCTCCGCCCCCCTGACGAGCATCACAAAAATCGACGCTCAAGTCAGAGGTGGCGAAACCCGA
CAGGACTATAAAGATACCAGGCGTTTCCCCCTGGAAGCTCCCTCGTGCGCTCTCCTGTTCCGACCCTGCCGCTTACCGGATACCTGTCCGCCTTTCTCCCTTCGGGAAGCGTGGCGCTTTCTCATAGCTCACG
CTGTAGGTATCTCAGTTCGGTGTAGGTCGTTCGCTCCAAGCTGGGCTGTGTGCACGAACCCCCCGTTCAGCCCGACCGCTGCGCCTTATCCGGTAACTATCGTCTTGAGTCCAACCCGGTAAGACACGACTTA
TCGCCACTGGCAGCAGCCACTGGTAACAGGATTAGCAGAGCGAGGTATGTAGGCGGTGCTACAGAGTTCTTGAAGTGGTGGCCTAACTACGGCTACACTAGAAGGACAGTATTTGGTATCTGCGCTCTGCTGA
AGCCAGTTACCTTCGGAAAAAGAGTTGGTAGCTCTTGATCCGGCAAACAAACCACCGCTGGTAGCGGTGGTTTTTTTGTTTGCAAGCAGCAGATTACGCGCAGAAAAAAAGGATCTCAAGAAGATCCTTTGAT
CTTTTCTACGGGGTCTGACGCTCAGTGGAACGAAAACTCACGTTAAGGGATTTTGGTCATGAGATTATCAAAAAGGATCTTCACCTAGATCCTTTTAAATTAAAAATGAAGTTTTAAATCAATCTAAAGTATA
TATGAGTAAACTTGGTCTGACAGTTACCAATGCTTAATCAGTGAGGCACCTATCTCAGCGATCTGTCTATTTCGTTCATCCATAGTTGCCTGACTCCCCGTCGTGTAGATAACTACGATACGGGAGGGCTTAC
CATCTGGCCCCAGTGCTGCAATGATACCGCGAGACCCACGCTCACCGGCTCCAGATTTATCAGCAATAAACCAGCCAGCCGGAAGGGCCGAGCGCAGAAGTGGTCCTGCAACTTTATCCGCCTCCATCCAGTC
TATTAATTGTTGCCGGGAAGCTAGAGTAAGTAGTTCGCCAGTTAATAGTTTGCGCAACGTTGTTGCCATTGCTACAGGCATCGTGGTGTCACGCTCGTCGTTTGGTATGGCTTCATTCAGCTCCGGTTCCCAA
CGATCAAGGCGAGTTACATGATCCCCCATGTTGTGCAAAAAAGCGGTTAGCTCCTTCGGTCCTCCGATCGTTGTCAGAAGTAAGTTGGCCGCAGTGTTATCACTCATGGTTATGGCAGCACTGCATAATTCTC
TTACTGTCATGCCATCCGTAAGATGCTTTTCTGTGACTGGTGAGTACTCAACCAAGTCATTCTGAGAATAGTGTATGCGGCGACCGAGTTGCTCTTGCCCGGCGTCAATACGGGATAATACCGCGCCACATAG
CAGAACTTTAAAAGTGCTCATCATTGGAAAACGTTCTTCGGGGCGAAAACTCTCAAGGATCTTACCGCTGTTGAGATCCAGTTCGATGTAACCCACTCGTGCACCCAACTGATCTTCAGCATCTTTTACTTTC
ACCAGCGTTTCTGGGTGAGCAAAAACAGGAAGGCAAAATGCCGCAAAAAAGGGAATAAGGGCGACACGGAAATGTTGAATACTCATACTCTTCCTTTTTCAATATTATTGAAGCATTTATCAGGGTTATTGTC
TCATGAGCGGATACATATTTGAATGTATTTAGAAAAATAAACAAATAGGGGTTCCGCGCACATTTCCCCGAAAAGTGC

b

>CD513B-cas9

acgcgtgtagtcttatgcaatactcttgtagtcttgcaacatggtaacgatgagttagcaacatgccttacaaggagagaaaaagcaccgtgcatgccgattggtggaagtaaggtggtacgatcgtgcctta
ttaggaaggcaacagacgggtctgacatggattggacgaaccactgaattgccgcattgcagagatattgtatttaagtgcctagctcgatacaataaacgggtctctctggttagaccagatctgagcctgg
gagctctctggctaactagggaacccactgcttaagcctcaataaagcttgccttgagtgcttcaagtagtgtgtgcccgtctgttgtgtgactctggtaactagagatccctcagacccttttagtcagtgt
ggaaaatctctagcagtggcgcccgaacagggacctgaaagcgaaagggaaaccagagctctctcgacgcaggactcggcttgctgaagcgcgcacggcaagaggcgaggggcggcgactggtgagtacgcca
aaaattttgactagcggaggctagaaggagagagatgggtgcgagagcgtcagtattaagcgggggagaattagatcgcgatgggaaaaaattcggttaaggccagggggaaagaaaaaatataaattaaaac
atatagtatgggcaagcagggagctagaacgattcgcagttaatcctggcctgttagaaacatcagaaggctgtagacaaatactgggacagctacaaccatcccttcagacaggatcagaagaacttagatc
attatataatacagtagcaaccctctattgtgtgcatcaaaggatagagataaaagacaccaaggaagctttagacaagatagaggaagagcaaaacaaaagtaagaccaccgcacagcaagcggccactgat
cttcagacctggaggaggagatatgagggacaattggagaagtgaattatataaatataaagtagtaaaaattgaaccattaggagtagcacccaccaaggcaaagagaagagtggtgcagagagaaaaaaga
gcagtgggaataggagctttgttccttgggttcttgggagcagcaggaagcactatgggcgcagcctcaatgacgctgacggtacaggccagacaattattgtctggtatagtgcagcagcagaacaatttgc
tgagggctattgaggcgcaacagcatctgttgcaactcacagtctggggcatcaagcagctccaggcaagaatcctggctgtggaaagatacctaaaggatcaacagctcctggggatttggggttgctctgg
aaaactcatttgcaccactgctgtgccttggaatgctagttggagtaataaatctctggaacagattggaatcacacgacctggatggagtgggacagagaaattaacaattacacaagcttaatacactcct
taattgaagaatcgcaaaaccagcaagaaaagaatgaacaagaattattggaattagataaatgggcaagtttgtggaattggtttaacataacaaattggctgtggtatataaaattattcataatgatagt
aggaggcttggtaggtttaagaatagtttttgctgtactttctatagtgaatagagttaggcagggatattcaccattatcgtttcagacccacctcccaaccccgaggggacccgacaggcccgaaggaata
gaagaagaaggtggagagagagacagagacagatccattcgattagtgaacggatctcgacggttaacttttaaaagaaaaggggggattggggggtacagtgcaggggaaagaatagtagacataatagcaa
cagacatacaaactaaagaattacaaaaacaaattacaaaaattcaaaattttatcgatactagtattatgcccagtacatgaccttatgggactttcctacttggcagtacatctacgtattagtcatcgct
attaccatggtgatgcggttttggcagtacatcaatgggcgtggatagcggtttgactcacggggatttccaagtctccaccccattgacgtcaatgggagtttgttttggcaccaaaatcaacgggactttc
caaaatgtcgtaacaactccgccccattgacgcaaatgggcggtaggcgtgtacggtgggaggtctatataagcagagctcgtttagtgaaccgtcagatcgcctggagacgccatccacgctgttttgacct
ccatagaagattctagaGTACCGCCACCATGGCCCCAAAGAAGAAGCGGAAGGTCGGTATCCACGGTGTCCCAGCAGCCATGGACAAGAAGTACTCCATTGGGCTCGATATCGGCACAAACAGCGTCGGCTGG
GCCGTCATTACGGACGAGTACAAGGTGCCGAGCAAAAAATTCAAAGTTCTGGGCAATACCGATCGCCACAGCATAAAGAAGAACCTCATTGGCGCCCTCCTGTTCGACTCCGGGGAGACGGCCGAAGCCACGC
GGCTCAAAAGAACAGCACGGCGCAGATATACCCGCAGAAAGAATCGGATCTGCTACCTGCAGGAGATCTTTAGTAATGAGATGGCTAAGGTGGATGACTCTTTCTTCCATAGGCTGGAGGAGTCCTTTTTGGT
GGAGGAGGATAAAAAGCACGAGCGCCACCCAATCTTTGGCAATATCGTGGACGAGGTGGCGTACCATGAAAAGTACCCAACCATATATCATCTGAGGAAGAAGCTTGTAGACAGTACTGATAAGGCTGACTTG
CGGTTGATCTATCTCGCGCTGGCGCATATGATCAAATTTCGGGGACACTTCCTCATCGAGGGGGACCTGAACCCAGACAACAGCGATGTCGACAAACTCTTTATCCAACTGGTTCAGACTTACAATCAGCTTT
TCGAAGAGAACCCGATCAACGCATCCGGAGTTGACGCCAAAGCAATCCTGAGCGCTAGGCTGTCCAAATCCCGGCGGCTCGAAAACCTCATCGCACAGCTCCCTGGGGAGAAGAAGAACGGCCTGTTTGGTAA
TCTTATCGCCCTGTCACTCGGGCTGACCCCCAACTTTAAATCTAACTTCGACCTGGCCGAAGATGCCAAGCTTCAACTGAGCAAAGACACCTACGATGATGATCTCGACAATCTGCTGGCCCAGATCGGCGAC
CAGTACGCAGACCTTTTTTTGGCGGCAAAGAACCTGTCAGACGCCATTCTGCTGAGTGATATTCTGCGAGTGAACACGGAGATCACCAAAGCTCCGCTGAGCGCTAGTATGATCAAGCGCTATGATGAGCACC
ACCAAGACTTGACTTTGCTGAAGGCCCTTGTCAGACAGCAACTGCCTGAGAAGTACAAGGAAATTTTCTTCGATCAGTCTAAAAATGGCTACGCCGGATACATTGACGGCGGAGCAAGCCAGGAGGAATTTTA
CAAATTTATTAAGCCCATCTTGGAAAAAATGGACGGCACCGAGGAGCTGCTGGTAAAGCTTAACAGAGAAGATCTGTTGCGCAAACAGCGCACTTTCGACAATGGAAGCATCCCCCACCAGATTCACCTGGGC
GAACTGCACGCTATCCTCAGGCGGCAAGAGGATTTCTACCCCTTTTTGAAAGATAACAGGGAAAAGATTGAGAAAATCCTCACATTTCGGATACCCTACTATGTAGGCCCCCTCGCCCGGGGAAATTCCAGAT
TCGCGTGGATGACTCGCAAATCAGAAGAGACCATCACTCCCTGGAACTTCGAGGAAGTCGTGGATAAGGGGGCCTCTGCCCAGTCCTTCATCGAAAGGATGACTAACTTTGATAAAAATCTGCCTAACGAAAA
GGTGCTTCCTAAACACTCTCTGCTGTACGAGTACTTCACAGTTTATAACGAGCTCACCAAGGTCAAATACGTCACAGAAGGGATGAGAAAGCCAGCATTCCTGTCTGGAGAGCAGAAGAAAGCTATCGTGGAC
CTCCTCTTCAAGACGAACCGGAAAGTTACCGTGAAACAGCTCAAAGAAGACTATTTCAAAAAGATTGAATGTTTCGACTCTGTTGAAATCAGCGGAGTGGAGGATCGCTTCAACGCATCCCTGGGAACGTATC
ACGATCTCCTGAAAATCATTAAAGACAAGGACTTCCTGGACAATGAGGAGAACGAGGACATTCTTGAGGACATTGTCCTCACCCTTACGTTGTTTGAAGATAGGGAGATGATTGAAGAACGCTTGAAAACTTA
CGCTCATCTCTTCGACGACAAAGTCATGAAACAGCTCAAGAGGCGCCGATATACAGGATGGGGGCGGCTGTCAAGAAAACTGATCAATGGGATCCGAGACAAGCAGAGTGGAAAGACAATCCTGGATTTTCTT
AAGTCCGATGGATTTGCCAACCGGAACTTCATGCAGTTGATCCATGATGACTCTCTCACCTTTAAGGAGGACATCCAGAAAGCACAAGTTTCTGGCCAGGGGGACAGTCTTCACGAGCACATCGCTAATCTTG
CAGGTAGCCCAGCTATCAAAAAGGGAATACTGCAGACCGTTAAGGTCGTGGATGAACTCGTCAAAGTAATGGGAAGGCATAAGCCCGAGAATATCGTTATCGAGATGGCCCGAGAGAACCAAACTACCCAGAA
GGGACAGAAGAACAGTAGGGAAAGGATGAAGAGGATTGAAGAGGGTATAAAAGAACTGGGGTCCCAAATCCTTAAGGAACACCCAGTTGAAAACACCCAGCTTCAGAATGAGAAGCTCTACCTGTACTACCTG
CAGAACGGCAGGGACATGTACGTGGATCAGGAACTGGACATCAATCGGCTCTCCGACTACGACGTGGATCATATCGTGCCCCAGTCTTTTCTCAAAGATGATTCTATTGATAATAAAGTGTTGACAAGATCCG
ATAAAAATAGAGGGAAGAGTGATAACGTCCCCTCAGAAGAAGTTGTCAAGAAAATGAAAAATTATTGGCGGCAGCTGCTGAACGCCAAACTGATCACACAACGGAAGTTCGATAATCTGACTAAGGCTGAACG
AGGTGGCCTGTCTGAGTTGGATAAAGCCGGCTTCATCAAAAGGCAGCTTGTTGAGACACGCCAGATCACCAAGCACGTGGCCCAAATTCTCGATTCACGCATGAACACCAAGTACGATGAAAATGACAAACTG
ATTCGAGAGGTGAAAGTTATTACTCTGAAGTCTAAGCTGGTCTCAGATTTCAGAAAGGACTTTCAGTTTTATAAGGTGAGAGAGATCAACAATTACCACCATGCGCATGATGCCTACCTGAATGCAGTGGTAG
GCACTGCACTTATCAAAAAATATCCCAAGCTTGAATCTGAATTTGTTTACGGAGACTATAAAGTGTACGATGTTAGGAAAATGATCGCAAAGTCTGAGCAGGAAATAGGCAAGGCCACCGCTAAGTACTTCTT
TTACAGCAATATTATGAATTTTTTCAAGACCGAGATTACACTGGCCAATGGAGAGATTCGGAAGCGACCACTTATCGAAACAAACGGAGAAACAGGAGAAATCGTGTGGGACAAGGGTAGGGATTTCGCGACA
GTCCGGAAGGTCCTGTCCATGCCGCAGGTGAACATCGTTAAAAAGACCGAAGTACAGACCGGAGGCTTCTCCAAGGAAAGTATCCTCCCGAAAAGGAACAGCGACAAGCTGATCGCACGCAAAAAAGATTGGG
ACCCCAAGAAATACGGCGGATTCGATTCTCCTACAGTCGCTTACAGTGTACTGGTTGTGGCCAAAGTGGAGAAAGGGAAGTCTAAAAAACTCAAAAGCGTCAAGGAACTGCTGGGCATCACAATCATGGAGCG
ATCAAGCTTCGAAAAAAACCCCATCGACTTTCTCGAGGCGAAAGGATATAAAGAGGTCAAAAAAGACCTCATCATTAAGCTTCCCAAGTACTCTCTCTTTGAGCTTGAAAACGGCCGGAAACGAATGCTCGCT
AGTGCGGGCGAGCTGCAGAAAGGTAACGAGCTGGCACTGCCCTCTAAATACGTTAATTTCTTGTATCTGGCCAGCCACTATGAAAAGCTCAAAGGGTCTCCCGAAGATAATGAGCAGAAGCAGCTGTTCGTGG
AACAACACAAACACTACCTTGATGAGATCATCGAGCAAATAAGCGAATTCTCCAAAAGAGTGATCCTCGCCGACGCTAACCTCGATAAGGTGCTTTCTGCTTACAATAAGCACAGGGATAAGCCCATCAGGGA
GCAGGCAGAAAACATTATCCACTTGTTTACTCTGACCAACTTGGGCGCGCCTGCAGCCTTCAAGTACTTCGACACCACCATAGACAGAAAGCGGTACACCTCTACAAAGGAGGTCCTGGACGCCACACTGATT
CATCAGTCAATTACGGGGCTCTATGAAACAAGAATCGACCTCTCTCAGCTCGGTGGAGACAAGCGTCCTGCTGCTACTAAGAAAGCTGGTCAAGCTAAGAAAAAGAAACCAAAGAAGAAGCGGAAGGTCTAAT
CTAGTGACTGACTAGGATCTGGTTACCACTAAACCAGCCTCAAGAACACCCGAATGGAGTCTCTAAGCTACATAATACCAACTTACACTTTACAAAATGTTGTCCCCCAAAATGTAGCCATTCGTATCTGCTC
CTAATAAAAAGAAAGTTTCTTCACATTCTAAAAAAAAAAAAAAAAAAAAAAAAAAAAAAAAAAAACCCCCCCCCCCTGCAGGTCGACTCTAGAGGATCCCCGGGCGAGCTCGAATTAATTCGTATTCTATAGTGT
CACCTAAATCCAATTCACTGGCGCggccgcaaggatctgcgatcgctccggtgcccgtcagtgggcagagcgcacatcgcccacagtccccgagaagttggggggaggggtcggcaattgaacgggtgcctag
agaaggtggcgcggggtaaactgggaaagtgatgtcgtgtactggctccgcctttttcccgagggtgggggagaaccgtatataagtgcagtagtcgccgtgaacgttctttttcgcaacgggtttgccgcca
gaacacagctgaagcttcgaggggctcgcatctctccttcacgcgcccgccgccctacctgaggccgccatccacgccggttgagtcgcgttctgccgcctcccgcctgtggtgcctcctgaactgcgtccgc
cgtctaggtaagtttaaagctcaggtcgagaccgggcctttgtccggcgctcccttggagcctacctagactcagccggctctccacgctttgcctgaccctgcttgctcaactctacgtctttgtttcgttt
tctgttctgcgccgttacagatccaagctgtgaccggcgcctacgctagacgccaccatggagagcgacgagagcggcctgcccgccatggagatcgagtgccgcatcaccggcaccctgaacggcgtggagt
tcgagctggtgggcggcggagagggcacccccaagcagggccgcatgaccaacaagatgaagagcaccaaaggcgccctgaccttcagcccctacctgctgagccacgtgatgggctacggcttctaccactt
cggcacctaccccagcggctacgagaaccccttcctgcacgccatcaacaacggcggctacaccaacacccgcatcgagaagtacgaggacggcggcgtgctgcacgtgagcttcagctaccgctacgaggcc
ggccgcgtgatcggcgacttcaaggtggtgggcaccggcttccccgaggacagcgtgatcttcaccgacaagatcatccgcagcaacgccaccgtggagcacctgcaccccatgggcgataacgtgctggtgg
gcagcttcgcccgcaccttcagcctgcgcgacggcggctactacagcttcgtggtggacagccacatgcacttcaagagcgccatccaccccagcatcctgcagaacggggcccccatgttcgccttccgccg
cgtggaggagctgcacagcaacaccgagctgggcatcgtggagtaccagcacgccttcaagacccccatcgccttcgccagatcccgcgctcagtcgtccaattctgccgtggacggcaccgccggacccggc
tccaccggatctcgcGAGGGCAGAGGAAGTCTTCTAACATGCGGTGACGTGGAGGAGAATCCCGGCCCTatgaccgagtacaagcccacggtgcgcctcgccacccgcgacgacgtccccagggccgtacgca
ccctcgccgccgcgttcgccgactaccccgccacgcgccacaccgtcgatccggaccgccacatcgagcgggtcaccgagctgcaagaactcttcctcacgcgcgtcgggctcgacatcggcaaggtgtgggt
cgcggacgacggcgccgcggtggcggtctggaccacgccggagagcgtcgaagcgggggcggtgttcgccgagatcggcccgcgcatggccgagttgagcggttcccggctggccgcgcagcaacagatggaa
ggcctcctggcgccgcaccggcccaaggagcccgcgtggttcctggccaccgtcggcgtctcgcccgaccaccagggcaagggtctgggcagcgccgtcgtgctccccggagtggaggcggccgagcgcgccg
gggtgcccgccttcctggagacctccgcgccccgcaacctccccttctacgagcggctcggcttcaccgtcaccgccgacgtcgaggtgcccgaaggaccgcgcacctggtgcatgacccgcaagcccggtgc
ctgaaatcaacctctggattacaaaatttgtgaaagattgactggtattcttaactatgttgctccttttacgctatgtggatacgctgctttaatgcctttgtatcatgctattgcttcccgtatggctttc
attttctcctccttgtataaatcctggttgctgtctctttatgaggagttgtggcccgttgtcaggcaacgtggcgtggtgtgcactgtgtttgctgacgcaacccccactggttggggcattgccaccacct
gtcagctcctttccgggactttcgctttccccctccctattgccacggcggaactcatcgccgcctgccttgcccgctgctggacaggggctcggctgttgggcactgacaattccgtggtgttgtcgggaa
gctgacgtcctttccatggctgctcgcctgtgttgccacctggattctgcgcgggacgtccttctgctacgtcccttcggccctcaatccagcggaccttccttcccgcggcctgctgccggctctgcggcct
cttccgcGtctCcgccttcgccctcagacgagtcggatctccctttggccgcctccccgcctggtacctttaagaccaatgacttacaaggcagctgtagatcttagccactttttaaaagaaaaggggggac
tggaagggctaattcactcccaacgaaaataagatctgcttttgcttgtactgggtctctctggttagaccagatctgagcctgggagctctctggctaactagggaacccactgcttaagcctcaataaag
cttgccttgagtgcttcaagtagtgtgtgcccgtctgttgtgtgactctggtaactagagatccctcagacccttttagtcagtgtggaaaatctctagcagtagtagttcatgtcatcttattattcagtat
ttataacttgcaaagaaatgaatatcagagagtgagaggaacttgtttattgcagcttataatggttacaaataaagcaatagcatcacaaatttcacaaataaagcatttttttcactgcattctagttgtg
gtttgtccaaactcatcaatgtatcttatcatgtctggctctagctatcccgcccctaactccgcccagttccgcccattctccgccccatggctgactaattttttttatttatgcagaggccgaggccgcc
tcggcctctgagctattccagaagtagtgaggaggcttttttggaggcctagacttttgcagagacggcccaaattcgtaatcatggtcatagctgtttcctgtgtgaaattgttatccgctcacaattccac
acaacatacgagccggaagcataaagtgtaaagcctggggtgcctaatgagtgagctaactcacattaattgcgttgcgctcactgcccgctttccagtcgggaaacctgtcgtgccagctgcattaatgaat
cggccaacgcgcggggagaggcggtttgcgtattgggcgctcttccgcttcctcgctcactgactcgctgcgctcggtcgttcggctgcggcgagcggtatcagctcactcaaaggcggtaatacggttatcc
acagaatcaggggataacgcaggaaagaacatgtgagcaaaaggccagcaaaaggccaggaaccgtaaaaaggccgcgttgctggcgtttttccataggctccgcccccctgacgagcatcacaaaaatcgac
gctcaagtcagaggtggcgaaacccgacaggactataaagataccaggcgtttccccctggaagctccctcgtgcgctctcctgttccgaccctgccgcttaccggatacctgtccgcctttctcccttcggg
aagcgtggcgctttctcatagctcacgctgtaggtatctcagttcggtgtaggtcgttcgctccaagctgggctgtgtgcacgaaccccccgttcagcccgaccgctgcgccttatccggtaactatcgtctt
gagtccaacccggtaagacacgacttatcgccactggcagcagccactggtaacaggattagcagagcgaggtatgtaggcggtgctacagagttcttgaagtggtggcctaactacggctacactagaagga
cagtatttggtatctgcgctctgctgaagccagttaccttcggaaaaagagttggtagctcttgatccggcaaacaaaccaccgctggtagcggtggtttttttgtttgcaagcagcagattacgcgcagaaa
aaaaggatctcaagaagatcctttgatcttttctacggggtctgacgctcagtggaacgaaaactcacgttaagggattttggtcatgagattatcaaaaaggatcttcacctagatccttttaaattaaaaa
tgaagttttaaatcaatctaaagtatatatgagtaaacttggtctgacagttaccaatgcttaatcagtgaggcacctatctcagcgatctgtctatttcgttcatccatagttgcctgactccccgtcgtgt
agataactacgatacgggagggcttaccatctggccccagtgctgcaatgataccgcgagacccacgctcaccggctccagatttatcagcaataaaccagccagccggaagggccgagcgcagaagtggtcc
tgcaactttatccgcctccatccagtctattaattgttgccgggaagctagagtaagtagttcgccagttaatagtttgcgcaacgttgttgccattgctacaggcatcgtggtgtcacgctcgtcgtttggt
atggcttcattcagctccggttcccaacgatcaaggcgagttacatgatcccccatgttgtgcaaaaaagcggttagctccttcggtcctccgatcgttgtcagaagtaagttggccgcagtgttatcactca
tggttatggcagcactgcataattctcttactgtcatgccatccgtaagatgcttttctgtgactggtgagtactcaaccaagtcattctgagaatagtgtatgcggcgaccgagttgctcttgcccggcgtc
aatacgggataataccgcgccacatagcagaactttaaaagtgctcatcattggaaaacgttcttcggggcgaaaactctcaaggatcttaccgctgttgagatccagttcgatgtaacccactcgtgcaccc
aactgatcttcagcatcttttactttcaccagcgtttctgggtgagcaaaaacaggaaggcaaaatgccgcaaaaaagggaataagggcgacacggaaatgttgaatactcatactcttcctttttcaatatt
attgaagcatttatcagggttattgtctcatgagcggatacatatttgaatgtatttagaaaaataaacaaataggggttccgcgcacatttccccgaaaagtgccacctgacgtctaagaaaccattattat
catgacattaacctataaaaataggcgtatcacgaggccctttcgtctcgcgcgtttcggtgatgacggtgaaaacctctgacacatgcagctcccggagacggtcacagcttgtctgtaagcggatgccggg
agcagacaagcccgtcagggcgcgtcagcgggtgttggcgggtgtcggggctggcttaactatgcggcatcagagcagattgtactgagagtgcaccatatgcggtgtgaaataccgcacagatgcgtaagga
gaaaataccgcatcaggcgccattcgccattcaggctgcgcaactgttgggaagggcgatcggtgcgggcctcttcgctattacgccagctggcgaaagggggatgtgctgcaaggcgattaagttgggtaac
gccagggttttcccagtcacgacgttgtaaaacgacggccagtgccaagctg

Figure S6. DNA sequences of plasmids used in this study. (a) PSK-u6+gRNA. Yellow highlight shows U6 promoter + gRNA; bold letters with underlining indicate scaffold of sgRNA. (b) CD513B-cas9. Purple, green, and blue highlights indicate cassettes of Cas9, *EGFP*, and ampicillin-resistance gene, respectively.
Figure S3, related to Figure 3

Supplemental Table S1: The primers used in this study

Name	5′-sequence-3′	Note
spacer sequence	CAGTGATAATTTCTGGGTTA	pegRNA, PE3
3' extension sequence	TTGCTATTGGGTTAACCCAGAAATTATC	
nick sgRNA	AATAAAAGCAGAATGGTAGC	Nick sgRNA, PE3
T7+spacer-F	caccGAAATTAATACGACTCACTATAGCAGTGATAATTTCTGGGTTAGTTTT	Construction of IVS-II-654_site pegRNA plasmid
T7+spacer-R	CTCTAAAACTAACCCAGAAATTATCACTGCTATAGTGAGTCGTATTAATTTC	
3' extension-F	GTGCTTGCTATTGGGTTAACCCAGAAATTATC	
3' extension-R	AAAAGATAATTTCTGGGTTAACCCAATAGCAA	
T7+nick sgRNA-F	tttGGAAATTAATACGACTCACTATAGAATAAAAGCAGAATGGTAGCgt	Construction of IVS-II-654_site Nick sgRNA plasmid
T7+nick sgRNA-R	taaaacGCTACCATTCTGCTTTTATTCTATAGTGAGTCGTATTAATTTC	
10533-WT-F	GATCCTATTGCCATGCCCTA	genotyping of Hbb^{th-4}/Hbb^{+} and Hbb^{+}/Hbb^{+}
10534WT-R	CTGTGCAGGCATGGTATGAC	
10535-MT-F	ACTCCTAAGCCAGTGCCAGA	
10536-MT-R	TGGTGTCTGTTTGAGGTTGC	
654-F	GACCAAATCAGGGTAATTTTGC	genome sequencing for checking prime edits
654-R	GGCCTAGCTTGGACTCAGAA	
GAPDH-RT-F	GCAGTGGCAAAGTGGAGATT	RT-PCR and sequencing
GAPDH-Rt-R	GTCTTCTGGGTGGCAGTGAT	
654-RT-F	CCTTTAGTGATGGCCTGGC	
654-RT-R	GCCACCACTTTCTGATAGGC	
sgRNA-F	tttgCAGTGATAATTTCTGGGTTAgt	Construction of the plasmid for off-target analysis of spacer sequence of pegRNA
sgRNA-R	taaaacTAACCCAGAAATTATCACTG	
nick sgRNA-F	tttgAATAAAAGCAGAATGGTAGCgt	Construction of the plasmid for off-target analysis of nick sgRNA of PE3
nick sgRNA-R	taaaacGCTACCATTCTGCTTTTATT	
NotI-HR-U6-sgRNA-F	GATCGCAGATCCTTGCGGCCGCCCGCTCTAGAGATCCGAC	Amplification of sgRNAs for construction of Cas9-sgRNA-EGFP
NotI-HR-U6-sgRNA-R	TCCAATTCACTGGCGCGGCCACTATAGGGCGAATTGGGTACC	
Sp-OT-1-F	ACACTCTTTCCCTACACGACGCTCTTCCGATCTNNNNATTATGGCAGCATGGCTCTGT	
Sp-OT-1-R	ACTGGAGTTCAGACGTGTGCTCTTCCGATCTGACACTCCACCCAACCCTCT	
Sp-OT-2-F	ACACTCTTTCCCTACACGACGCTCTTCCGATCTNNNNAATCCCTCTGGGAGCACTTT	
Sp-OT-2-R	ACTGGAGTTCAGACGTGTGCTCTTCCGATCTGCTTCATTTTTGAAGCCAACAGT	
Sp-OT-3-F	ACACTCTTTCCCTACACGACGCTCTTCCGATCTNNNNGGGCACATTTTGACACTAACTT	
Sp-OT-3-R	ACTGGAGTTCAGACGTGTGCTCTTCCGATCTAGCCCTTAAAGAATGGTCGC	
Ni-OT-1-F	ACACTCTTTCCCTACACGACGCTCTTCCGATCTNNNNCCCTTCTTTCTGGGAAGGTGAG	
Ni-OT-1-R	ACTGGAGTTCAGACGTGTGCTCTTCCGATCTGACTTTGTGGGGAAAGGGCAT	
Ni-OT-2-F	ACACTCTTTCCCTACACGACGCTCTTCCGATCTNNNNAGTTCCAGTATCAACCTGGCT	
Ni-OT-2-R	ACTGGAGTTCAGACGTGTGCTCTTCCGATCTTGGCTCTGGTTGTCATTTCCA	
Ni-OT-3-F	ACACTCTTTCCCTACACGACGCTCTTCCGATCTNNNNAGCTGTACCTCTCGAGCATTT	
Ni-OT-3-R	ACTGGAGTTCAGACGTGTGCTCTTCCGATCTCTCAACACCAAGGGCTGCTC	

Supplemental Table S2: Raw data of hematologic analyses

Samples	WBC	LYM#	MID#	GRAN#	RBC	HGB	HCT	MCV	MCH	MCHC	RDW_CV	RDW_SD	PLT	PDW	PCT
WT	7.10	4.30	1.70	1.10	16.15	295.00	88.80	55.00	18.20	332.00	11.40	20.00	733.00	3.80	0.30
	4.80	2.90	1.10	0.70	13.95	247.00	75.60	54.20	17.70	326.00	11.60	20.00	730.00	3.60	0.29
	4.70	2.90	1.10	0.70	13.97	247.00	75.80	54.30	17.60	325.00	11.50	20.00	724.00	3.60	0.30
	4.70	2.90	1.10	0.70	13.53	237.00	73.30	54.20	17.50	323.00	11.60	18.20	704.00	3.50	0.28
	4.20	2.60	0.90	0.70	13.07	228.00	70.80	54.20	17.40	322.00	11.60	20.00	713.00	3.60	0.29
	6.10	3.60	1.70	0.80	15.77	286.00	91.10	57.80	18.10	313.00	11.70	20.00	780.00	3.80	0.31
	6.30	3.80	1.60	0.90	15.89	287.00	92.00	57.90	18.00	311.00	11.70	21.80	761.00	3.60	0.31
	5.70	3.40	1.50	0.80	14.52	263.00	83.70	57.70	18.10	314.00	10.80	20.00	738.00	3.80	0.30
	5.80	3.60	1.50	0.60	14.66	263.00	84.20	57.50	17.90	312.00	11.70	20.00	755.00	3.80	0.33
beta	20.80	13.50	5.30	2.00	11.18	167.00	48.10	43.10	14.90	347.00	23.40	25.40	1264.00	21.00	0.54
	19.60	13.20	4.90	1.50	11.68	168.00	50.50	43.30	14.30	332.00	23.40	23.60	1989.00	21.00	0.52
	18.20	12.00	4.70	1.50	11.54	165.00	49.70	43.10	14.20	331.00	23.50	27.30	1420.00	21.00	0.61
	17.50	11.70	4.20	1.60	11.15	161.00	47.90	43.00	14.40	336.00	23.50	25.40	1392.00	21.00	0.61
	17.60	11.40	4.40	1.80	11.08	162.00	47.80	43.20	14.60	338.00	23.40	25.40	1289.00	21.00	0.55
	19.90	13.40	5.00	1.50	11.91	174.00	53.30	44.80	14.60	326.00	23.60	27.30	1472.00	21.00	0.63
	21.60	14.40	5.50	1.70	12.10	177.00	54.50	45.10	14.60	324.00	22.50	29.10	1528.00	21.00	0.65
	17.90	11.80	4.50	1.60	11.06	161.00	49.40	44.70	14.50	325.00	22.60	25.40	1389.00	21.00	0.59
	17.40	11.10	4.90	1.40	10.82	171.00	48.00	44.40	15.80	356.00	22.80	27.30	1361.00	21.00	0.59
beta-PE	8.30	4.60	2.50	1.20	16.71	294.00	93.00	55.70	17.50	316.00	12.10	21.80	720.00	3.60	0.30
	8.50	4.60	2.30	1.60	16.61	295.00	92.60	55.80	17.70	318.00	12.00	20.00	698.00	3.80	0.28
	6.70	3.70	2.00	1.00	14.43	248.00	79.60	55.20	17.10	311.00	11.30	20.00	682.00	3.60	0.27
	7.90	4.20	2.20	1.50	15.15	266.00	83.70	55.30	17.50	317.00	11.30	20.00	676.00	3.60	0.27
	5.70	2.90	1.70	1.20	11.72	192.00	60.40	51.60	16.30	317.00	11.20	18.20	547.00	3.60	0.22
	7.20	3.90	1.90	1.40	14.31	240.00	74.90	52.40	16.70	320.00	11.00	20.00	568.00	3.60	0.23
	6.00	3.40	1.60	0.90	13.59	224.00	70.90	52.20	16.40	315.00	11.00	18.20	559.00	3.60	0.23
	6.60	3.70	1.70	1.20	13.74	229.00	71.80	52.30	16.60	318.00	11.00	20.00	583.00	3.60	0.23
	6.50	3.90	1.60	1.10	16.46	286.00	87.40	53.10	17.30	327.00	10.80	20.00	665.00	3.60	0.27
	9.60	5.00	2.80	1.80	16.77	288.00	97.70	53.60	17.60	330.00	11.70	21.80	666.00	3.60	0.27
beta-PE F_1	6.00	4.20	1.40	0.40	15.36	268.00	86.30	56.20	17.40	310.00	11.10	20.00	565.00	3.90	0.24
	7.60	5.20	1.80	0.60	17.14	265.00	97.30	56.80	17.70	313.00	11.80	21.80	544.00	4.10	0.24
	7.40	4.80	1.90	0.60	15.21	266.00	85.60	56.30	17.40	310.00	11.90	21.80	743.00	4.10	0.32
	7.40	5.00	1.80	0.60	16.86	295.00	95.20	56.50	17.40	309.00	11.90	21.80	551.00	4.10	0.24

	5.70	3.80	1.40	0.60	12.74	212.00	67.00	52.60	16.60	316.00	11.90	18.20	497.00	3.80	0.21
	6.90	4.50	1.70	0.60	15.34	263.00	81.70	53.30	17.10	321.00	11.70	20.00	572.00	3.90	0.25
	6.50	4.50	1.50	0.50	15.16	262.00	80.80	53.30	17.20	324.00	10.80	20.00	496.00	3.80	0.21
	6.60	4.50	1.60	0.50	15.29	264.00	81.80	53.50	17.20	322.00	11.70	20.00	473.00	3.90	0.20
	6.60	4.50	1.60	0.40	14.32	246.00	76.00	53.10	17.10	323.00	11.70	20.00	682.00	3.80	0.29
	6.20	4.20	1.50	0.40	14.39	245.00	76.20	53.00	17.00	321.00	10.80	20.00	647.00	3.80	0.28

Supplemental Table S3: Raw data of heart and spleen coefficient (organ mass divided by body mass)

Gender	Name	body weight (g)	heart weight (g)	spleen weight (g)
WT-male	No.1	24.175	0.130	0.073
	No.2	24.188	0.128	0.066
	No.3	24.436	0.116	0.081
	No.4	26.125	0.128	0.081
	No.5	28.249	0.144	0.08
	No.6	25.312	0.124	0.088
WT-female	No.1	18.784	0.115	0.063
	No.2	19.431	0.110	0.087
	No.3	20.218	0.117	0.077
	No.4	19.896	0.120	0.093
	No.5	18.077	0.101	0.081
	No.6	19.541	0.097	0.077
	No.7	17.108	0.089	
	No.8	17.236	0.096	
beta-male	No.1	20.856	0.158	0.335
	No.2	23.244	0.177	0.276
	No.3	21.784	0.159	0.283
	No.4	20.038	0.155	0.278
	No.5	26.757	0.213	0.354
	No.6	25.134	0.187	0.313
beta-female	No.1	23.820	0.169	0.298
	No.2	18.630	0.142	0.265
	No.3	18.670	0.139	0.273
	No.4	21.824	0.147	0.276
	No.5	18.769	0.113	0.348
	No.6	19.863	0.122	0.346
	No.7	20.303	0.122	
beta-PE male	17#	24.040	0.146	0.080
	18#	24.103	0.136	0.089
beta-PE F_1 male	No.1	25.421	0.139	0.077
	No.2	24.891	0.136	0.078
beta-PE F_1 female	No.1	19.436	0.131	0.079
	No.2	20.125	0.131	0.072
	No.3	22.531	0.137	0.066

	No.4	26.090	0.131	0.077

Supplemental Table S4: Raw data of visceral coefficient (organ mass divided by body mass)

gender	name	body weight (g)	heart weight (g)	liver weight (g)	spleen weight (g)	lung weight (g)	kidney weight (g)	ovary and uterus weight (g)	testis weight (g)	epididymis weight (g)	thymus weight (g)	brain weight (g)
beta-male	No.1	20.856	0.158	1.328	0.335	0.146	0.326		0.164	0.016	0.077	0.411
	No.2	23.244	0.177	1.334	0.276	0.177	0.390		0.224	0.027	0.057	0.440
	No.3	21.784	0.159	1.340	0.283	0.167	0.363		0.197	0.019	0.064	0.454
	No.4	20.038	0.155	1.120	0.278	0.137	0.294		0.188	0.024	0.059	0.417
	No.5	26.757	0.213	1.745	0.354	0.299	0.454		0.235	0.046	0.072	0.461
	No.6	25.134	0.187	1.605	0.313	0.206	0.373		0.231	0.041	0.067	0.447
beta-female	No.1	23.820	0.169	1.506	0.298	0.182	0.355	0.089			0.095	0.450
	No.2	18.630	0.142	1.153	0.265	0.162	0.276	0.104			0.084	0.425
	No.3	18.670	0.139	1.221	0.273	0.139	0.287	0.069			0.084	0.429
	No.4	21.824	0.147	1.427	0.276	0.145	0.312	0.087			0.076	0.457
	No.5	18.769	0.113	1.143	0.348	0.22	0.26	0.074			0.093	0.431
	No.6	19.863	0.122	1.246	0.346	0.175	0.285	0.102			0.078	0.447
	No.7	20.303	0.122					0.102				
WT-male	No.1	24.175	0.130	1.500	0.073	0.169	0.395		0.186	0.019	0.045	0.499
	No.2	24.188	0.128	1.255	0.066	0.189	0.427		0.196	0.021	0.046	0.486
	No.3	24.436	0.116	1.461	0.081	0.166	0.397		0.183	0.020	0.050	0.452
	No.4	26.125	0.128	1.538	0.081	0.187	0.401		0.175	0.028	0.052	0.471
	No.5	28.249	0.144	1.695	0.08	0.277	0.478		0.37	0.03	0.073	0.453
	No.6	25.312	0.124	1.649	0.088	0.284	0.403		0.211	0.022	0.056	0.43
WT-female	No.1	18.784	0.115	1.104	0.063	0.165	0.305	0.071			0.070	0.476
	No.2	19.431	0.110	1.222	0.087	0.175	0.313	0.099			0.065	0.476
	No.3	20.218	0.117	1.141	0.077	0.197	0.337	0.086			0.042	0.497
	No.4	19.896	0.120	1.276	0.093	0.189	0.335	0.113			0.067	0.491
	No.5	18.077	0.101	1.030	0.081	0.197	0.266	0.056			0.095	0.447
	No.6	19.541	0.097	1.090	0.077	0.16	0.25	0.15			0.097	0.448
	No.7	17.108	0.089					0.077				
	No.8	17.236	0.096					0.072				

Supplemental Table S5: Raw data of deep sequencing for off-target analysis

**Peg OT-1**					
location	A	C	G	T	ref base
1	97.34	0.14	0.12	2.41	A
2	0.53	0.08	0.21	99.19	T
3	3.06	0.15	0.24	96.55	T
4	95.46	0.16	0.65	3.73	A
5	0.25	0.33	4.68	94.75	T
6	0.26	0.9	98.67	0.18	G
7	0.86	4.91	94.13	0.1	G
8	5.15	93.76	0.98	0.11	C
9	93.51	0.92	5.32	0.25	A
10	1.06	5.45	93.19	0.3	G
11	5.51	92.94	0.56	0.99	C
12	92.78	0.36	1.13	5.74	A
13	0.32	0.29	6.73	92.65	T
14	0.25	0.99	98.28	0.49	G
15	0.1	6.26	92.55	1.09	G
16	0.06	93.31	0.37	6.26	C
17	0.11	6.17	0.27	93.44	T
18	0.08	92.45	1.01	6.46	C
19	0.18	0.24	6.16	93.42	T
20	0.16	0.22	92.49	7.14	G
21	0.22	0.57	0.56	98.65	T
22	0.64	7.91	1.53	89.92	T
23	1.02	7.77	6.51	84.7	T
24	8.67	0.15	90.23	0.95	G
25	5.78	0.58	86.16	7.48	G
26	85.49	0.58	13.43	0.5	A
27	5.85	7.48	85.83	0.84	G
28	84.56	0.41	7.18	7.84	A
29	1.04	0.28	97.64	1.04	G
30	7.52	0.53	85.98	5.97	G
31	0.68	0.31	13.38	85.63	T
32	7.52	0.8	85.54	6.13	G
33	0.56	7.76	5.85	85.84	T

34	0.24	1.01	84.84	13.92	G
35	0.3	5.6	0.89	93.21	T
36	0.58	84.37	1.79	13.26	C
37	0.34	0.44	14.69	84.53	T
38	0.49	0.24	97.9	1.38	G
39	1.08	0.35	92.69	5.88	G
40	8.5	0.64	6.38	84.48	T
41	6.63	1.13	91.86	0.38	G
42	89.92	9.07	0.53	0.47	A
43	84.84	13.24	0.32	1.6	A
44	1.01	85.74	0.43	12.82	C
45	1.12	13.51	0.55	84.82	T
46	6.33	91.62	1.39	0.65	C
47	92.43	0.49	6.53	0.55	A
48	5.91	0.98	92.65	0.46	G
49	84.99	8.52	5.87	0.63	A
50	8.45	5.77	84.78	1	G
51	5.63	84.74	1.78	7.86	C
52	84.96	1.27	13.12	0.66	A
53	8	6.29	85.15	0.56	G
54	0.45	89.85	8.35	1.35	C
55	0.36	84.68	8.43	6.53	C
56	1.31	0.42	6.01	92.26	T
57	5.72	0.18	85.87	8.23	g
58	84.81	0.28	13.83	1.09	A
59	0.62	0.88	92.65	5.84	G
60	1.48	7.4	6.1	85.02	T
61	13.01	0.27	84.78	1.94	G
62	84.62	0.83	1.25	13.29	A
63	1.33	8.08	5.75	84.83	T
64	5.98	7.53	84.47	2.03	g
65	84.82	0.42	0.73	14.03	A
66	7.62	0.59	0.32	91.47	T
67	0.56	0.9	0.36	98.18	T
68	0.59	5.6	0.87	92.93	T
69	7.44	84.54	1.93	6.09	C
70	0.65	0.79	13.96	84.6	T

71	0.29	7.6	90.96	1.16	G
72	0.42	0.68	90.26	8.65	G
73	0.87	7.54	85.17	6.43	G
74	8.67	0.31	1.05	89.98	T
75	6.77	0.88	7.86	84.5	T
76	90.29	7.91	1.32	0.48	A
77	92.19	0.42	6.59	0.8	A
78	0.8	0.88	90.65	7.67	G
79	0.99	8.3	90.42	0.29	G
80	5.85	8.24	85.47	0.44	G
81	84.73	8.91	6.05	0.32	A
82	2.06	13.19	84.58	0.17	G
83	14.11	84.53	1.09	0.26	C
84	90.05	0.69	8.84	0.42	A
85	85.71	0.44	13.51	0.34	A
86	6.05	1.03	92.34	0.58	G
87	84.93	6.04	7.78	1.25	A
88	7.68	84.82	0.91	6.59	C
89	0.82	0.6	7.54	91.04	T
90	7.92	1.2	1.06	89.82	T
91	1.54	6.21	7.6	84.65	T
92	13.44	85.24	0.93	0.4	C
93	85.23	6.06	8.28	0.44	A
94	5.92	85.47	8.35	0.27	C
95	84.6	7.07	7.81	0.52	A
96	1.36	97.11	0.36	1.17	C
97	6.42	84.9	0.64	8.04	C
98	89.83	7.64	0.75	1.78	A
99	84.63	0.29	1.04	14.03	A
100	0.44	0.83	5.89	92.84	T
101	0.62	7.59	85.14	6.66	G
102	0.63	0.33	5.89	93.15	T
103	1.7	0.35	84.32	13.63	G
104	6.7	1.06	0.42	91.82	T
105	90.45	8.05	0.31	1.19	A
106	85.66	8.27	0.32	5.75	A
107	7.46	7.52	0.32	84.7	T

108	97.45	0.32	0.32	1.91	A
109	85.32	0.49	0.22	13.96	A
110	7.82	1.37	0.54	90.27	T
111	1.04	12.97	0.64	85.35	T
112	8.42	84.88	0.37	6.33	C
113	5.85	0.73	1.36	92.07	T
114	85.47	0.94	5.76	7.84	A
115	5.84	8.98	84.78	0.41	G
116	84.72	13.61	1.34	0.33	A
117	0.41	91.9	6.8	0.89	C
118	0.25	1.63	90.25	7.88	G
119	0.87	13.09	84.93	1.11	G
120	8.53	84.53	1.1	5.85	C
121	8.39	0.51	5.75	85.35	T
122	8.62	0.53	85.01	5.84	G
123	5.95	0.96	8.75	84.35	T
124	84.39	8.54	6.1	0.97	A
125	0.74	7.14	84.52	7.6	G
126	0.38	97.88	0.62	1.12	C
127	0.71	93.12	0.39	5.78	C
128	0.94	13.16	0.75	85.15	T
129	8.7	84.45	0.94	5.91	C
130	6.87	0.9	7.78	84.46	T
131	97.31	1.53	0.73	0.43	A
132	84.48	6.97	7.83	0.73	A
133	0.35	90.81	0.65	8.19	C
134	0.41	90.79	0.79	8.01	C
135	0.3	90.21	8.02	1.46	C
136	0.33	85.06	7.66	6.95	C
137	0.39	7.73	0.85	91.04	T
138	1.42	0.22	1.03	97.33	T
139	5.98	0.45	8.61	84.96	T
140	84.59	1.17	6.29	7.95	A
141	0.86	5.77	92.27	1.1	G
142	7.54	84.56	1.51	6.39	C
143	0.35	0.4	6.83	92.42	T
144	0.51	0.23	91.15	8.11	G

145	0.34	0.45	97.72	1.49	G
146	0.73	1.13	91.94	6.2	G
147	7.57	6.28	0.63	85.53	T
148	0.73	92.03	0.34	6.9	C
149	7.78	0.66	0.74	90.82	T
150	0.83	1.25	7.99	89.93	T
151	7.54	6.5	0.3	85.66	T
152	0.43	89.91	0.55	9.11	C
153	1.06	84.75	0.55	13.64	C
154	5.6	1.18	1.01	92.22	T
155	84.37	5.9	8.03	1.7	A
156	0.39	84.75	8.11	6.75	C
157	0.65	0.52	7.6	91.24	T
158	0.55	0.69	0.91	97.85	T
159	0.72	7.87	2.27	89.15	T
160	7.78	1.87	5.73	84.61	T
161	1.56	13.11	84.42	0.9	G
162	13.15	84.83	0.5	1.51	C
163	84.7	1.49	0.7	13.1	A
164	0.64	7.04	7.57	84.75	T
165	0.23	97.93	0.5	1.34	C
166	0.62	92.7	0.2	6.48	C
167	0.99	8.68	0.64	89.69	T
168	8.44	6.05	0.47	85.03	T
169	5.99	85.41	0.9	7.7	C
170	85.19	6.65	7.63	0.53	A
171	5.92	93.21	0.36	0.51	C
172	84.78	14.13	0.96	0.12	A
173	1.8	90.22	7.81	0.16	C
174	13.66	85.45	0.64	0.25	C
175	93.05	5.84	0.66	0.45	A
176	13.19	84.99	1.3	0.52	C
177	84.71	8.17	6.1	1.01	A
178	1.46	7.49	85.35	5.71	G
179	9.02	0.25	6.04	84.69	T
180	13.87	0.27	84.59	1.27	G
181	90.35	0.71	0.66	8.27	A

182	85.67	7.94	0.4	6	A
183	6.6	7.74	0.52	85.15	T
184	90.41	0.17	1.56	7.86	A
185	85	0.26	13.61	1.13	A
186	0.73	0.91	92.31	6.05	G
187	1.27	5.65	0.98	92.11	T
188	6.47	84.79	8.14	0.6	C
189	90.38	0.84	7.77	1	A
190	85.22	7.98	1.04	5.76	A
191	1.62	7.53	5.83	85.02	T
192	13.09	0.3	85.59	1.02	G
193	85.64	0.19	5.88	8.29	A
194	8.72	0.16	85.41	5.71	G
195	6.59	0.37	8.2	84.84	T
196	91.04	0.86	7.52	0.58	A
197	92.92	5.65	0.34	1.09	A
198	8.47	84.91	0.39	6.22	C
199	5.74	0.54	1.48	92.23	T
200	85	0.99	13.52	0.5	A
201	1.09	5.66	92.62	0.62	G
202	6.14	84.93	7.71	1.21	C
203	92.34	0.36	0.22	7.08	A
204	0.9	0.15	0.32	98.62	T
205	7.51	0.7	1.07	90.73	T
206	0.76	7.61	5.71	85.91	T
207	7.75	0.96	84.93	6.35	G
208	1.06	5.8	0.56	92.58	T
209	5.89	85.04	1.37	7.7	C
210	86.22	0.58	12.93	0.28	A
211	13.41	1.55	84.81	0.23	G
212	85.59	13.81	0.42	0.18	A
213	8.41	90.98	0.35	0.26	C
214	5.76	93.07	0.69	0.48	C
215	84.86	5.92	7.53	1.69	A
216	0.48	85.37	0.36	13.8	C
217	0.26	8.09	1.05	90.6	T
218	0.55	7.93	5.61	85.91	T

219	1.47	7.5	84.96	6.07	G
220	13.07	0.72	0.25	85.96	T
221	85.41	7.61	0.29	6.7	A
222	7.64	0.93	0.67	90.75	T
223	0.68	5.63	7.6	86.09	T
224	7.5	84.84	0.65	7.01	C
225	0.19	0.3	8.06	91.45	T
226	0.44	0.11	7.88	91.57	T
227	1	0.04	7.59	91.36	T
228	5.84	0.05	0.37	93.74	T
229	85.69	0.06	0.85	13.4	A
230	5.83	0.08	8.11	85.99	T
231	84.99	0.14	7.98	6.9	A
232	0.37	0.09	7.51	92.02	T
233	0.27	0.3	0.81	98.62	T
234	0.51	0.84	8.03	90.62	T
235	1.69	5.63	7.65	85.04	T
236	13.77	84.88	1.06	0.3	C
237	90.49	0.33	8.42	0.76	A
238	85.38	0.11	7.21	7.3	A
239	1.35	0.21	97.7	0.74	G
240	6.54	0.79	85.45	7.23	G
241	91.42	7.31	1.16	0.1	A
242	93.38	0.12	6.36	0.14	A
243	6.51	0.04	93.16	0.29	G
244	93.14	0.19	6.37	0.3	A
245	6.56	0.06	92.37	1.01	G
246	93.24	0.04	0.66	6.06	A
247	6.43	0.01	1.25	92.3	T
248	93.17	0.01	6.41	0.41	A
249	6.3	0.02	93.47	0.21	G
250	92.29	0.12	7.42	0.17	A
251	0.21	0.14	99.25	0.41	G
252	0.08	0.09	98.68	1.15	G
253	0.21	0.02	92.8	6.97	G
254	0.13	0.11	1.5	98.27	T
255	0.15	0.02	7.46	92.36	T

256	0.16	0.11	99.26	0.48	G
257	0.14	0.11	98.74	1.01	G
258	0.14	0.1	93.08	6.68	G
259	0.33	0.03	8.24	91.4	T
260	1.06	0.06	98.48	0.4	G
261	7.62	0.07	91.98	0.33	G
262	90.33	0.07	8.33	1.28	A
263	0.25	0.2	91.16	8.39	G
264	0.02	0.4	8.55	91.04	T
265	0.12	1.27	89.94	8.67	G
266	0.01	8.74	0.3	90.96	T
267	0.04	99.58	0.04	0.34	C
**sequence**					**length**
ATTATGGCAGCATGGCTCTGTTTGGAGAGGTGTGTCTGGTGAACTCAGAGCAGCCTgAGTGATgATTTCTGGGTTAAGGGAGCAAGACTTTCACACCAATGTGTAATAATTCTAGACGGCTGTAGCCTCTAACCCCTTTAGCTGGGTCTTTCCTACTTTTGCATCCTTCACACCACAGTGAATAAGTCAATGAGTAACTAGCATTTGTCAGACCACTTGTATTCTTTTATATTTTCAAGGAAGAGATAGAGGGTTGGGTGGAGTGTC					267
**total reads**	**perfect reads**	**off-target editing ratio**			
3031940	3025287	0.22%			
**Peg OT-2**					
location	A	C	G	T	ref base
1	99.34	0.16	0.16	0.35	A
2	95.92	0.67	0.14	3.26	A
3	0.14	4.61	0.07	95.18	T
4	0.05	99.39	0.05	0.51	C
5	0.14	98.73	0.19	0.93	C
6	0.04	93.46	0.31	6.18	C
7	0.18	6.66	0.68	92.48	T
8	0.19	90.21	2.11	7.49	C
9	0.21	0.26	10.32	89.21	T
10	0.58	0.1	98.97	0.36	G
11	1.92	0.29	97.7	0.09	G
12	9.07	0.56	90.2	0.16	G
13	88.65	2.06	9.16	0.13	A
14	2.04	9.53	88.17	0.27	G
15	9.17	89.92	0.19	0.72	C
16	88.19	9.17	0.1	2.55	A

17	0.41	88.26	0.01	11.32	C
18	0.84	0.24	0.02	98.9	T
19	2.42	0.07	0.02	97.49	T
20	10.77	0.21	0.04	88.98	T
21	97.1	0.26	0.02	2.63	A
22	88.58	0.5	0.02	10.9	A
23	0.7	1.83	0.1	97.37	T
24	1.86	8.98	0.11	89.05	T
25	9.04	88.21	0.03	2.73	C
26	88.35	0.29	0.03	11.33	A
27	0.68	0.36	0.14	98.82	T
28	1.94	0.81	0.05	97.2	T
29	8.98	2.51	0.12	88.39	T
30	88.16	11.46	0.13	0.26	A
31	0.33	99.33	0.04	0.3	C
32	0.32	99.12	0.03	0.53	C
33	0.35	97.62	0.03	2	C
34	0.58	90.04	0.14	9.25	C
35	2.01	9.13	0.1	88.75	T
36	9.51	88.25	0.19	2.05	C
37	90.08	0.25	0.52	9.14	A
38	9.47	0.04	1.84	88.66	T
39	88.8	0.13	9.04	2.03	A
40	2.69	0.11	88.02	9.17	G
41	11.32	0.04	0.22	88.42	T
42	98.84	0.02	0.22	0.92	A
43	97.21	0.02	0.2	2.56	A
44	88.37	0.06	0.22	11.34	A
45	0.26	0.15	0.64	98.95	T
46	0.23	0.17	2.29	97.31	T
47	0.65	0.2	10.75	88.4	T
48	2	0.61	97	0.39	G
49	9.55	1.95	88.3	0.2	G
50	90.1	9	0.37	0.52	A
51	9.28	88.25	0.58	1.89	C
52	88.29	0.67	2	9.03	A
53	0.75	1.82	9.07	88.35	T

54	2.01	9.05	88.18	0.75	G
55	9.55	88.25	0.33	1.86	C
56	90.19	0.26	0.53	9.03	A
57	9.84	0.04	1.94	88.19	T
58	90.46	0.02	9.09	0.43	A
59	11.06	0.03	88.34	0.57	g
60	97.28	0.03	0.67	2.01	A
61	88.78	0.08	1.93	9.21	a
62	2.18	0.16	9.04	88.63	T
63	9.53	0.18	88.18	2.12	G
64	89.89	0.58	0.2	9.32	A
65	9.13	1.92	0.03	88.92	T
66	88.18	9.03	0.05	2.74	A
67	0.31	88.33	0.06	11.3	c
68	0.1	0.75	0.12	99.03	T
69	0.03	1.92	0.37	97.69	T
70	0.1	8.95	0.74	90.21	T
71	0.04	88.1	2.58	9.28	C
72	0.16	0.18	11.34	88.32	T
73	0.2	0.01	98.93	0.86	G
74	0.26	0.05	97.28	2.41	G
75	0.54	0.1	88.54	10.82	G
76	1.94	0.1	0.87	97.08	T
77	9.11	0.1	2.48	88.31	T
78	88.28	0.02	11.42	0.28	A
79	0.71	0.11	98.9	0.29	G
80	1.99	0.09	97.12	0.79	G
81	9.56	0.05	88.27	2.13	G
82	89.97	0.13	0.35	9.55	A
83	9.24	0.16	0.49	90.12	T
84	88.13	0.21	1.85	9.81	A
85	0.42	0.1	8.96	90.52	T
86	0.11	0.19	88.17	11.53	G
87	0.34	0.56	0.28	98.82	T
88	0.59	2.08	0.1	97.23	T
89	2.15	9.52	0.03	88.3	T
90	9.79	89.89	0.07	0.25	C

91	90.5	9.13	0.25	0.12	A
92	11.12	88.2	0.48	0.19	C
93	97.36	0.19	1.82	0.63	A
94	89.04	0.15	8.98	1.83	A
95	2.74	0.1	88.13	9.03	G
96	11.52	0.06	0.19	88.23	T
97	99.47	0.05	0.11	0.36	A
98	99.61	0.1	0.1	0.19	A
99	99.18	0.17	0.04	0.61	A
100	97.46	0.54	0.08	1.92	A
101	88.88	1.92	0.19	9.02	A
102	2.19	9.01	0.52	88.28	T
103	9.54	88.24	1.92	0.3	C
104	90.19	0.67	9.07	0.08	A
105	9.93	1.91	88.13	0.03	G
106	90.56	9.05	0.34	0.05	A
107	11.24	88.09	0.5	0.18	C
108	97.78	0.2	1.92	0.1	A
109	90.62	0.08	8.97	0.33	A
110	11.06	0.25	88.09	0.61	G
111	97.26	0.49	0.22	2.04	A
112	88.52	1.84	0.1	9.54	A
113	0.78	9.01	0.23	89.98	T
114	1.93	88.25	0.64	9.18	C
115	9.16	0.3	2.31	88.23	T
116	88.59	0.13	10.92	0.36	A
117	2.11	0.25	96.99	0.65	G
118	9.08	0.8	88.24	1.88	G
119	88.44	2.29	0.2	9.07	A
120	0.92	10.8	0.11	88.16	T
121	2.64	97.03	0.03	0.29	C
122	11.53	88.23	0.2	0.04	C
123	99.55	0.25	0.13	0.06	A
124	99.73	0.05	0.08	0.14	A
125	99.61	0.01	0.28	0.11	A
126	99.2	0.03	0.57	0.2	A
127	97.43	0.04	1.85	0.68	A

128	88.85	0.11	9.18	1.85	A
129	2.76	0.13	88.01	9.1	G
130	11.51	0.07	0.26	88.16	T
131	99.3	0.19	0.14	0.37	A
132	99.13	0.5	0.17	0.2	A
133	97.29	2	0.18	0.53	A
134	88.47	9.08	0.59	1.85	A
135	0.96	88.12	1.85	9.08	C
136	2.65	0.34	9.05	87.97	T
137	11.78	0.02	87.91	0.29	G
138	99.5	0.13	0.22	0.15	A
139	99.56	0.11	0.03	0.3	A
140	99.05	0.11	0.06	0.78	A
141	97.41	0.03	0.13	2.43	A
142	88.63	0.03	0.21	11.13	A
143	2.09	0.03	0.1	97.79	T
144	9.16	0.06	0.12	90.66	T
145	88.09	0.16	0.04	11.71	A
146	0.4	0.25	0.04	99.31	T
147	0.19	0.52	0.08	99.21	T
148	0.51	1.95	0.17	97.37	T
149	1.94	9.13	0.18	88.75	T
150	9.23	87.91	0.57	2.29	C
151	88.19	0.21	1.83	9.76	A
152	0.49	0.05	9.15	90.32	T
153	0.75	0.19	87.93	11.14	G
154	2.59	0.1	0.24	97.07	T
155	11.45	0.02	0.1	88.42	T
156	99.01	0.03	0.12	0.84	A
157	97.82	0.02	0.18	1.98	A
158	90.47	0.11	0.1	9.31	A
159	11.22	0.21	0.05	88.52	T
160	97.52	0.12	0.05	2.3	A
161	89.93	0.2	0.04	9.82	A
162	9.32	0.06	0.09	90.53	T
163	88.09	0.08	0.22	11.61	A
164	0.79	0.29	0.05	98.87	T

165	1.91	0.6	0.12	97.37	T
166	9.1	1.93	0.28	88.69	T
167	87.94	9.08	0.6	2.38	A
168	0.41	87.93	1.91	9.74	C
169	0.07	0.27	9.34	90.32	T
170	0.13	0.17	88.54	11.15	G
171	0.05	0.28	2.5	97.18	T
172	0.14	0.55	10.9	88.42	T
173	0.09	1.97	97.02	0.92	G
174	0.14	9.28	88.07	2.5	G
175	0.38	87.88	0.24	11.51	C
176	0.95	2.28	0.02	96.75	T
177	3.07	10.45	0.02	86.45	T
178	14.08	85.41	0.08	0.43	C
179	99.41	0.25	0.18	0.15	A
180	99.4	0.04	0.16	0.41	A
181	98.74	0.15	0.34	0.77	A
182	96.1	0.16	0.87	2.87	A
183	83.39	0.17	3	13.44	A
184	4.03	0.18	13.99	81.8	T
185	17.09	0.27	82.34	0.3	G
186	95.71	0.83	3.41	0.05	A
187	82.18	3.2	14.44	0.17	A
188	0.38	14.98	84.47	0.17	G
189	0.09	99.2	0.48	0.23	C
**sequence**					**length**
AATCCCTCTGGGAGCACTTTAATTCATTTACCCCTCATAGTAAATTTGGACATGCATAgAaTGATAcTTTCTGGGTTAGGGATATGTTTCACAAGTAAAAATCAGACAAGAATCTAGGATCCAAAAAAGTAAAACTGAAAAATTATTTTCATGTTAAATAATATTTACTGTTGGCTTCAAAAATGAAGC					189
**total reads**	**perfect reads**	**off-target editing ratio**			
3063921	3054409	0.31%			
**Peg OT-3**					
location	A	C	G	T	ref base
1	0.19	0.09	99.46	0.26	G
2	0.14	0.17	99.4	0.29	G
3	0.2	1.58	98.09	0.13	G
4	1.62	98.13	0.14	0.1	C

5	97.98	1.83	0.04	0.15	A
6	1.94	97.6	0.05	0.41	C
7	97.32	0.16	0.27	2.26	A
8	0.22	0.1	0.28	99.4	T
9	0.11	0.17	0.2	99.52	T
10	0.27	0.19	0.39	99.14	T
11	0.47	0.37	3.19	95.97	T
12	3.61	0.47	95.58	0.34	G
13	95.92	3.71	0.19	0.19	A
14	3.83	95.77	0.06	0.34	C
15	95.43	3.99	0.04	0.54	A
16	0.74	95.44	0.1	3.72	C
17	4.24	0.32	0.1	95.34	T
18	98.91	0.6	0.02	0.47	A
19	95.46	3.88	0.03	0.63	A
20	0.31	95.36	0.05	4.28	C
21	0.69	0.2	0.03	99.08	T
22	3.83	0.13	0.05	95.99	T
23	95.26	0.02	0.09	4.63	A
24	0.22	0.12	0.19	99.47	T
25	0.1	0.15	0.57	99.18	T
26	0.15	0.04	4.11	95.7	T
27	0.17	0.14	98.93	0.75	G
28	0.54	0.21	95.49	3.77	G
29	3.85	0.02	0.65	95.48	T
30	95.88	0.02	3.74	0.36	A
31	4.29	0.05	95.38	0.28	G
32	98.95	0.08	0.53	0.44	A
33	95.62	0.07	0.6	3.72	A
34	0.65	0.19	3.79	95.37	T
35	3.81	0.16	95.63	0.39	G
36	95.65	0.18	3.98	0.18	A
37	3.96	0.48	95.25	0.3	G
38	95.26	3.87	0.23	0.64	A
39	0.33	95.78	0.08	3.81	C
40	0.05	4	0.14	95.81	T
41	0.27	95.29	0.45	3.99	C

42	0.21	0.32	3.77	95.7	T
43	0.29	0.57	95.22	3.93	G
44	0.6	3.77	0.18	95.44	T
45	3.83	95.38	0.02	0.78	C
46	95.28	0.82	0.14	3.76	A
47	0.31	4.26	0.03	95.4	T
48	0.5	99.11	0.07	0.32	C
49	3.75	95.51	0.04	0.7	C
50	95.37	0.61	0.04	3.97	A
51	0.62	3.72	0.22	95.44	T
52	3.83	95.24	0.14	0.8	C
53	95.37	0.27	0.06	4.29	A
54	0.23	0.05	0.21	99.51	T
55	0.15	0.08	0.17	99.6	T
56	0.47	0.05	0.14	99.34	T
57	3.71	0.08	0.46	95.75	T
58	95.34	0.23	3.71	0.72	A
59	0.3	0.54	95.32	3.84	G
60	0.58	3.69	0.3	95.43	T
61	3.73	95.35	0.48	0.44	C
62	95.28	0.23	3.69	0.8	A
63	0.26	0.32	95.27	4.15	G
64	0.17	0.44	0.25	99.15	T
65	0.55	3.68	0.11	95.65	T
66	3.73	95.33	0.28	0.66	C
67	95.38	0.22	0.59	3.82	A
68	0.27	0.16	4.28	95.29	T
69	0.18	0.07	99.39	0.35	G
70	0.56	0.15	99.13	0.16	G
71	4.15	0.16	95.53	0.16	G
72	99.19	0.43	0.25	0.13	A
73	95.96	3.67	0.12	0.25	A
74	4.01	95.23	0.25	0.51	C
75	95.35	0.29	0.47	3.9	A
76	0.65	0.04	3.7	95.61	T
77	3.77	0.13	95.33	0.77	G
78	95.38	0.16	0.23	4.22	A

79	0.76	0.12	0.09	99.02	T
80	3.83	0.55	0.21	95.41	T
81	95.8	3.7	0.26	0.24	A
82	4.01	95.22	0.56	0.21	C
83	95.78	0.23	3.79	0.2	A
84	3.99	0.04	95.22	0.74	G
85	95.67	0.07	0.19	4.07	A
86	3.85	0.28	0.07	95.81	T
87	95.25	0.24	0.04	4.48	A
88	0.39	0.52	0.04	99.06	T
89	0.05	3.83	0.12	96	T
90	0.26	95.71	0.01	4.02	C
91	0.22	4.07	0.02	95.69	T
92	0.23	95.71	0.12	3.95	C
93	0.6	3.98	0.02	95.41	T
94	3.92	95.29	0.04	0.75	C
95	95.35	0.31	0.14	4.2	A
96	0.39	0.53	0.05	99.03	T
97	0.06	3.9	0.14	95.9	T
98	0.1	95.84	0.06	4	C
99	0.19	3.86	0.22	95.72	T
100	0.09	95.25	0.63	4.04	C
101	0.12	0.41	3.69	95.78	T
102	0.39	0.02	95.21	4.37	G
103	0.57	0.16	0.2	99.07	T
104	4.26	0.05	0.13	95.56	T
105	99.13	0.12	0.05	0.71	A
106	95.87	0.04	0.02	4.07	A
107	4.02	0.06	0.1	95.82	T
108	95.44	0.14	0.03	4.39	A
109	0.46	0.16	0.01	99.37	T
110	0.68	0.13	0.06	99.13	T
111	3.78	0.47	0.01	95.74	T
112	95.48	3.75	0.11	0.67	A
113	0.75	95.33	0.11	3.8	C
114	3.88	0.65	0.1	95.36	T
115	95.39	3.81	0.04	0.76	A

116	0.45	95.3	0.12	4.13	C
117	0.48	0.48	0.04	99	T
118	3.87	0.67	0.07	95.39	T
119	95.39	4.22	0.15	0.24	A
120	0.73	99.03	0.15	0.09	C
121	4.16	95.5	0.13	0.21	C
122	98.94	0.64	0.15	0.27	A
123	95.53	3.69	0.26	0.52	A
124	0.78	95.24	0.18	3.81	C
125	3.9	0.3	0.16	95.64	T
126	95.46	0.07	0.49	3.98	A
127	0.64	0.09	4.01	95.26	T
128	3.78	0.24	95.61	0.37	G
129	95.33	0.55	3.93	0.19	A
130	0.29	3.7	95.41	0.6	G
131	0.09	95.33	0.45	4.13	C
132	0.21	0.25	0.56	98.98	T
133	0.12	0.3	4.11	95.48	T
134	0.25	0.61	98.9	0.25	G
135	0.45	3.94	95.5	0.11	G
136	3.71	95.64	0.36	0.29	C
137	95.25	3.95	0.29	0.51	A
138	0.25	95.34	0.48	3.93	C
139	0.23	0.22	3.79	95.75	t
140	0.21	0.04	95.74	4.01	G
141	0.53	0.24	3.9	95.33	T
142	3.99	0.03	95.24	0.74	G
143	95.95	0.02	0.25	3.79	A
144	4.28	0.05	0.27	95.41	T
145	99.02	0.14	0.48	0.35	A
146	95.4	0.06	3.85	0.7	A
147	0.3	0.24	95.25	4.21	g
148	0.13	0.48	0.28	99.11	T
149	0.02	3.7	0.4	95.88	T
150	0.06	95.32	0.7	3.92	C
151	0.21	0.19	4.3	95.3	T
152	0.23	0.04	99.3	0.43	G

153	0.28	0.04	99.19	0.49	G
154	0.68	0.1	95.42	3.8	G
155	4.25	0.02	0.27	95.45	T
156	98.9	0.04	0.29	0.77	a
157	95.39	0.02	0.58	4.01	A
158	0.24	0.03	4.19	95.54	T
159	0.03	0.06	99.15	0.75	G
160	0.08	0.18	95.93	3.81	G
161	0.16	0.15	4.34	95.35	T
162	0.09	0.69	99	0.23	G
163	0.28	3.85	95.8	0.06	G
164	0.6	95.42	3.87	0.12	C
165	3.84	0.73	95.21	0.22	G
166	95.31	4.23	0.21	0.25	A
167	0.65	98.85	0.04	0.46	C
168	3.86	95.24	0.23	0.67	C
169	94.95	0.32	0.03	4.7	A
170	0.22	0.68	0.07	99.02	T
171	0.16	4.26	0.14	95.44	T
172	0.12	94.36	0.07	5.45	C
173	0.3	0.23	0.1	99.37	T
174	0.88	0.07	0.24	98.81	T
175	7.06	0.06	0.25	92.63	T
176	98.4	0.17	1.14	0.3	A
177	91.04	0.17	8.56	0.23	A
178	0.26	0.26	99.17	0.31	G
179	0.07	1.21	98.34	0.38	G
180	0.09	8.83	89.66	1.43	G
181	0.18	90.23	0.35	9.23	C
182	0.21	0.28	0.12	99.39	T
**sequence**					**length**
GGGCACATTTTGACACTAACTTATTTGGTAGAATGAGACTCTGTCATCCATCATTTTAGTCAGTTCATGGGAACATGATTACAGATATTCTCTCATTCTCTGTTAATATTTACTACTTACCAACTATGAGCTTGGCACtGTGATAAgTTCTGGGTaATGGTGGCGACCATTCTTTAAGGGCT					182
**total reads**	**perfect reads**	**off-target editing ratio**			
3238212	3232790	0.17%			
**Ni OT-1**					

location	A	C	G	T	ref base
1	0.11	99.78	0.02	0.09	C
2	0.02	99.72	0.06	0.2	C
3	0.02	97.82	0.02	2.14	C
4	0.05	0.36	0.02	99.57	T
5	0.04	2.81	0.06	97.09	T
6	0.05	96.17	0.05	3.74	C
7	0.07	0.22	0.09	99.62	T
8	0.05	0.62	0.18	99.15	T
9	0.03	5.29	0.3	94.38	T
10	0.12	92.51	1.25	6.13	C
11	0.13	0.18	8.19	91.5	T
12	0.44	0.05	99.32	0.19	G
13	1.63	0.08	98.25	0.04	G
14	9.55	0.03	90.34	0.07	G
15	97.87	0.08	1.93	0.11	A
16	89.1	0.07	10.47	0.36	A
17	0.36	0.04	98.05	1.55	G
18	0.43	0.07	90.41	9.09	G
19	1.5	0.09	9.5	88.91	T
20	9.14	0.09	90.19	0.58	G
21	88.85	0.05	9.23	1.86	A
22	0.53	0.15	88.79	10.53	G
23	1.54	0.42	0.19	97.85	T
24	9.13	1.82	0.05	89	T
25	88.95	10.69	0.08	0.28	A
26	1.65	97.78	0.16	0.41	C
27	9.15	88.92	0.45	1.48	C
28	88.81	0.22	1.9	9.07	A
29	0.31	0.07	10.91	88.71	T
30	0.47	0.03	99.24	0.26	G
31	1.91	0.04	97.94	0.11	G
32	10.87	0.08	88.91	0.14	G
33	99.29	0.12	0.21	0.38	A
34	98.02	0.38	0.05	1.56	A
35	89.3	1.51	0.08	9.12	A
36	2.01	9.16	0.1	88.73	T

37	10.65	89	0.06	0.29	C
38	97.85	1.62	0.16	0.37	A
39	89.06	9.11	0.35	1.48	A
40	0.62	88.76	1.55	9.07	C
41	1.94	0.18	9.17	88.71	T
42	10.59	0.11	89.1	0.2	G
43	97.9	0.35	1.67	0.08	A
44	89.05	1.72	9.14	0.09	A
45	1.99	9.07	88.74	0.21	G
46	10.59	88.73	0.22	0.47	C
47	97.81	0.22	0.17	1.81	A
48	88.86	0.22	0.39	10.53	A
49	0.58	0.04	1.64	97.73	T
50	1.64	0.02	9.42	88.92	T
51	9.44	0.02	90.32	0.22	G
52	90.26	0.01	9.68	0.05	A
53	9.35	0.03	90.51	0.1	G
54	89.15	0.02	10.77	0.06	A
55	2.06	0.06	97.86	0.02	G
56	10.91	0.04	88.98	0.07	G
57	99.37	0.04	0.53	0.06	A
58	98.33	0.05	1.61	0.02	A
59	90.67	0.05	9.2	0.07	A
60	10.83	0.02	89.04	0.11	G
61	98.1	0.04	1.79	0.06	A
62	90.35	0.02	9.53	0.09	A
63	9.39	0.04	90.48	0.1	G
64	89.16	0.01	10.7	0.12	A
65	2.04	0.04	97.63	0.3	G
66	10.9	0.07	88.89	0.14	G
67	99.28	0.05	0.51	0.16	A
68	97.96	0.08	1.57	0.38	A
69	89.19	0.02	9.28	1.51	A
70	1.7	0.04	89.14	9.12	G
71	9.15	0.13	1.98	88.75	T
72	88.9	0.32	10.56	0.22	A
73	0.67	1.48	97.7	0.15	G

74	1.98	9.07	88.71	0.24	G
75	10.98	88.64	0.19	0.2	C
76	99.41	0.15	0.06	0.38	A
77	98.38	0.05	0.06	1.51	A
78	90.77	0.06	0.07	9.1	A
79	11.12	0.07	0.06	88.75	T
80	99.61	0.07	0.14	0.19	A
81	99.53	0.03	0.4	0.03	A
82	98.36	0.03	1.57	0.05	A
83	90.64	0.07	9.24	0.05	A
84	10.82	0.05	89	0.13	G
85	98.2	0.03	1.65	0.12	a
86	90.6	0.04	9.15	0.2	A
87	10.71	0.01	88.86	0.41	G
88	97.81	0.04	0.6	1.55	A
89	88.9	0.04	1.84	9.22	A
90	0.36	0.02	10.56	89.06	T
91	0.46	0.02	97.74	1.78	G
92	1.65	0.02	88.84	9.49	G
93	9.54	0.02	0.21	90.23	T
94	90.54	0.02	0.21	9.24	A
95	10.79	0.05	0.44	88.71	t
96	97.92	0.04	1.83	0.2	a
97	89.21	0.06	10.65	0.08	A
98	2.04	0.05	97.82	0.09	G
99	10.74	0.04	89.14	0.09	G
100	98.12	0.08	1.68	0.12	A
101	90.36	0.13	9.14	0.37	A
102	9.46	0.33	88.67	1.53	G
103	89.16	1.52	0.17	9.16	A
104	2.09	9.16	0.06	88.69	T
105	11.1	88.71	0.04	0.15	C
106	99.6	0.22	0.11	0.07	A
107	99.46	0.14	0.36	0.04	A
108	97.99	0.41	1.52	0.08	A
109	88.98	1.8	9.11	0.11	A
110	0.63	10.59	88.7	0.09	G

111	2	97.72	0.14	0.13	C
112	10.97	88.81	0.02	0.2	C
113	99.39	0.17	0.02	0.42	A
114	98.26	0.04	0.02	1.67	A
115	90.34	0.03	0.03	9.61	A
116	9.29	0.02	0.07	90.61	T
117	88.81	0.05	0.05	11.09	A
118	0.51	0.1	0.02	99.36	T
119	1.57	0.06	0.04	98.33	T
120	9.24	0.05	0.1	90.6	T
121	89.05	0.12	0.05	10.78	A
122	1.73	0.36	0.05	97.87	T
123	9.49	1.51	0.07	88.93	T
124	90.23	9.13	0.06	0.58	A
125	9.65	88.71	0.05	1.6	C
126	90.21	0.26	0.06	9.48	A
127	9.35	0.39	0.09	90.18	T
128	89	1.55	0.16	9.29	A
129	1.71	9.21	0.36	88.72	T
130	9.24	88.99	1.52	0.26	C
131	88.99	1.65	9.16	0.2	A
132	1.7	9.2	88.69	0.41	G
133	9.16	89.03	0.19	1.63	C
134	88.72	1.64	0.12	9.51	A
135	0.19	9.18	0.37	90.26	T
136	0.1	88.68	1.59	9.62	C
137	0.18	0.19	9.45	90.19	T
138	0.41	0.07	90.13	9.39	G
139	1.65	0.05	9.26	89.05	T
140	9.45	0.07	88.72	1.77	G
141	90.17	0.07	0.2	9.56	A
142	9.29	0.03	0.15	90.54	T
143	88.73	0.04	0.39	10.84	A
144	0.2	0.04	1.63	98.13	T
145	0.11	0.06	9.46	90.37	T
146	0.11	0.05	90.25	9.59	G
147	0.14	0.1	9.57	90.18	T

148	0.4	0.15	90.18	9.27	G
149	1.6	0.37	9.33	88.69	T
150	9.49	1.5	88.82	0.19	G
151	90.16	9.13	0.63	0.08	A
152	9.32	88.67	1.91	0.09	C
153	88.66	0.22	10.94	0.17	A
154	0.24	0.12	99.19	0.45	G
155	0.08	0.34	97.85	1.73	G
156	0.04	1.54	88.89	9.53	G
157	0.06	9.17	0.49	90.28	T
158	0.1	88.78	1.52	9.6	C
159	0.16	0.55	9.16	90.13	T
160	0.46	1.53	88.73	9.28	G
161	1.6	9.2	0.48	88.72	T
162	9.48	88.76	1.53	0.24	C
163	90.18	0.46	9.15	0.22	A
164	9.42	1.49	88.68	0.41	G
165	89.01	9.12	0.16	1.71	A
166	1.82	88.65	0.02	9.51	C
167	9.46	0.19	0.01	90.34	T
168	90.19	0.07	0.05	9.69	A
169	9.36	0.07	0.04	90.53	T
170	89	0.19	0.06	10.75	A
171	1.65	0.44	0.08	97.84	T
172	9.16	1.81	0.09	88.94	T
173	88.74	10.61	0.12	0.53	A
174	0.28	97.79	0.38	1.54	C
175	0.39	88.88	1.51	9.21	C
176	1.56	0.51	9.13	88.81	T
177	9.2	1.48	88.66	0.65	G
178	88.85	9.11	0.18	1.86	A
179	0.63	88.68	0.08	10.61	C
180	1.86	0.16	0.14	97.84	T
181	10.64	0.03	0.47	88.86	T
182	97.87	0.02	1.84	0.27	A
183	89.1	0.07	10.62	0.21	A
184	1.68	0.04	97.74	0.54	G

185	9.18	0.04	88.78	2	G
186	88.75	0.04	0.16	11.05	A
187	0.3	0.03	0.02	99.65	T
188	0.44	0.05	0.06	99.45	T
189	1.87	0.07	0.02	98.04	T
190	10.69	0.09	0.02	89.2	T
191	98.11	0.06	0.07	1.77	A
192	90.27	0.09	0.11	9.52	A
193	9.33	0.17	0.35	90.16	T
194	88.66	0.48	1.51	9.35	A
195	0.2	1.93	9.15	88.72	T
196	0.08	10.96	88.64	0.32	G
197	0.07	99.24	0.18	0.51	C
198	0.09	97.92	0.01	1.98	C
199	0.06	88.83	0.07	11.03	C
200	0.06	0.68	0.04	99.21	T
201	0.09	2.1	0.03	97.78	T
202	0.09	11.89	0.03	87.99	T
203	0.22	99.47	0.04	0.27	C
204	0.57	99.28	0.02	0.13	C
205	2.36	97.46	0.02	0.15	C
206	13.73	86.06	0.06	0.16	C
207	85.53	14.25	0.1	0.11	A
208	19.14	80.45	0.25	0.16	C
209	98.41	0.36	0.91	0.32	A
210	94.79	0.34	3.86	1.02	A
211	77.51	1.08	17.33	4.08	A
212	0.31	4.22	77.5	17.97	G
213	0.09	18.99	0.26	80.66	T
214	0.1	99.43	0.03	0.44	C
**sequence**					**length**
CCCTTCTTTCTGGGAAGGTGAGTTACCATGGGAAATCAACTGAAGCAATTGAGAGGAAAGAAGAGGAAAGTAGGCAAATAAAAGaAGAATGGTAtaAGGAAGATCAAAAGCCAAATATTTATTACATATCAGCATCTGTGATATTGTGTGACAGGGTCTGTCAGACTATATTACCTGACTTAAGGATTTTAATATGCCCTTTCCCCACAAAGTC					214
**total reads**	**perfect reads**	**off-target editing ratio**			
3536654	3525917	0.30%			
**Ni OT-2**					

location	A	C	G	T	ref base
1	98.29	0.19	1.31	0.21	A
2	0.43	0.09	97.75	1.73	G
3	0.05	0.36	0.33	99.26	T
4	0.21	2.24	0.03	97.51	T
5	0.28	99.12	0.14	0.46	C
6	2.77	96.77	0.34	0.13	C
7	96.1	0.4	3.12	0.38	A
8	0.8	0.14	95.65	3.41	G
9	3.64	0.19	0.35	95.81	T
10	95.2	0.5	0.13	4.16	A
11	0.92	4.26	0.07	94.75	T
12	4.91	94.59	0.07	0.43	C
13	98.7	1.06	0.13	0.11	A
14	94.38	5.26	0.19	0.17	A
15	0.44	98.67	0.22	0.67	C
16	0.07	94.06	0.79	5.08	C
17	0.12	0.54	5.68	93.66	T
18	0.14	0.74	98.59	0.53	G
19	0.23	5.09	93.88	0.8	G
20	0.67	93.61	0.46	5.26	C
21	5.19	0.41	0.02	94.38	T
22	93.63	0.17	0.1	6.1	A
23	0.47	0.62	0.02	98.89	T
24	0.23	5.08	0.07	94.61	T
25	0.77	93.63	0.11	5.49	C
26	5.72	0.53	0.04	93.72	T
27	98.63	0.7	0.12	0.55	A
28	94.03	5.11	0.2	0.66	A
29	0.42	93.56	0.73	5.29	C
30	0.16	0.38	5.22	94.25	T
31	0.65	0.05	93.69	5.61	G
32	5.21	0.02	0.63	94.14	T
33	93.76	0.06	0.68	5.5	A
34	1.14	0.09	5.17	93.6	T
35	5.76	0.01	93.69	0.54	G
36	98.73	0.09	1.08	0.11	A

37	94.56	0.04	5.3	0.1	A
38	5.52	0.01	94.38	0.09	G
39	93.8	0.07	6.1	0.03	A
40	1.13	0.12	98.65	0.09	G
41	5.74	0.05	94.1	0.1	G
42	98.7	0.02	1.13	0.15	A
43	94.02	0.2	5.15	0.63	A
44	1.11	0.06	93.68	5.15	G
45	5.23	0.02	1.04	93.71	T
46	94.17	0.1	5.15	0.59	A
47	5.53	0.04	93.57	0.86	G
48	93.64	0.04	0.48	5.84	A
49	0.57	0.02	0.02	99.38	T
50	0.66	0.06	0.09	99.2	T
51	5.11	0.06	0.16	94.66	T
52	93.66	0.01	0.25	6.08	A
53	0.48	0.01	0.7	98.81	T
54	0.14	0.07	5.14	94.64	T
55	0.15	0.14	93.64	6.07	G
56	0.68	0.06	0.43	98.83	T
57	5.23	0.02	0.14	94.6	T
58	94.25	0.05	0.22	5.48	A
59	5.55	0.03	0.67	93.75	T
60	93.63	0.04	5.12	1.21	A
61	0.58	0.09	93.62	5.71	G
62	0.75	0.17	0.42	98.66	T
63	5.11	0.8	0.09	94	T
64	93.61	5.66	0.18	0.55	A
65	0.46	98.6	0.26	0.68	C
66	0.14	93.96	0.78	5.13	C
67	0.24	0.42	5.7	93.64	T
68	0.69	0.05	98.78	0.47	G
69	5.14	0.11	94.59	0.16	G
70	93.67	0.02	5.63	0.67	A
71	0.52	0.08	94.23	5.17	G
72	0.81	0.05	5.48	93.67	T
73	5.28	0.07	93.68	0.97	G

74	94.28	0.16	0.44	5.13	A
75	5.58	0.74	0.05	93.63	T
76	94.3	5.27	0.03	0.4	A
77	5.67	94.25	0.05	0.03	C
78	93.77	6.05	0.15	0.04	A
79	1.13	98.69	0.07	0.11	C
80	5.86	93.93	0.11	0.1	C
81	99.36	0.38	0.05	0.21	A
82	99.16	0.03	0.14	0.67	A
83	94.56	0.1	0.05	5.28	A
84	5.51	0.08	0.06	94.35	T
85	93.7	0.18	0.03	6.1	A
86	0.45	0.69	0.1	98.77	T
87	0.21	5.12	0.13	94.54	T
88	0.72	93.62	0.17	5.49	C
89	5.2	0.37	0.67	93.77	T
90	93.61	0.03	5.18	1.18	A
91	0.47	0.01	93.76	5.76	G
92	0.23	0.06	1.05	98.65	T
93	0.7	0.02	5.18	94.1	T
94	5.18	0.1	93.68	1.04	G
95	93.65	0.04	1.04	5.27	A
96	0.49	0.07	5.14	94.29	T
97	0.09	0.07	93.78	6.06	G
98	0.18	0.06	1.16	98.6	T
99	0.32	0.04	5.73	93.91	T
100	0.83	0.09	98.63	0.45	G
101	5.83	0.16	93.93	0.08	G
102	98.78	0.6	0.49	0.13	A
103	94.64	5.09	0.16	0.11	A
104	6.13	93.55	0.17	0.16	C
105	98.75	0.41	0.63	0.21	A
106	94.05	0.04	5.16	0.76	A
107	0.58	0.03	93.66	5.73	G
108	0.37	0.04	1	98.59	T
109	0.87	0.1	5.08	93.95	T
110	5.77	0.17	93.58	0.48	G

111	98.67	0.66	0.43	0.24	A
112	94.06	5.22	0.05	0.68	A
113	1.1	93.56	0.09	5.25	C
114	5.27	0.45	0.13	94.15	T
115	93.64	0.22	0.66	5.47	A
116	0.54	0.73	5.13	93.6	T
117	0.27	5.73	93.5	0.49	G
118	1.04	98.37	0.47	0.12	C
119	5.96	93.75	0.1	0.19	C
120	98.8	0.42	0.08	0.7	A
121	94.62	0.03	0.06	5.29	A
122	5.58	0.07	0.13	94.22	T
123	94.22	0.04	0.2	5.54	A
124	5.56	0.14	0.68	93.62	T
125	93.66	0.64	5.19	0.51	A
126	0.67	5.16	93.22	0.95	G
127	1.02	93.43	0.4	5.16	C
128	5.88	0.42	0.08	93.61	T
129	99.39	0.07	0.05	0.49	A
130	99.63	0.06	0.09	0.22	A
131	99.27	0.03	0.05	0.65	A
132	94.74	0.06	0.1	5.1	A
133	6.08	0.28	0.05	93.59	T
134	98.79	0.69	0.01	0.51	A
135	94.08	5.2	0.02	0.7	A
136	1.25	93.6	0.04	5.1	C
137	5.87	0.44	0.04	93.65	T
138	99.33	0.1	0.09	0.48	A
139	99.67	0.07	0.02	0.24	A
140	99.07	0.19	0.08	0.66	A
141	93.97	0.74	0.08	5.21	A
142	0.58	5.19	0.05	94.18	T
143	0.7	93.67	0.06	5.57	C
144	5.18	1.02	0.1	93.69	T
145	93.63	5.24	0.13	1	A
146	0.51	94.24	0.15	5.11	C
147	0.22	6.07	0.06	93.65	T

148	0.87	98.49	0.24	0.4	C
149	5.33	93.85	0.65	0.18	C
150	94.26	0.43	5.16	0.15	A
151	6.14	0.08	93.38	0.4	G
152	98.57	0.18	0.39	0.86	A
153	93.86	0.26	0.09	5.8	A
154	0.46	0.65	0.31	98.58	T
155	0.16	5.09	0.22	94.53	T
156	0.2	93.6	0.68	5.52	C
157	0.74	0.43	5.18	93.65	T
158	5.73	0.08	93.65	0.53	G
159	98.46	0.13	1.14	0.27	A
160	93.79	0.17	5.78	0.27	A
161	0.44	0.06	98.62	0.88	G
162	0.09	0.18	94.41	5.33	G
163	0.3	0.08	5.49	94.13	T
164	0.84	0.05	93.5	5.61	G
165	5.75	0.2	0.46	93.59	T
166	98.57	0.77	0.09	0.57	A
167	93.79	5.14	0.2	0.87	A
168	0.47	93.56	0.81	5.16	C
169	0.11	0.42	5.86	93.61	T
170	0.25	0.05	99.2	0.5	G
171	0.62	0.08	98.92	0.38	G
172	5.19	0.08	94.54	0.19	G
173	93.55	0.15	6.06	0.24	A
174	0.44	0.15	98.45	0.96	G
175	0.22	0.08	93.8	5.9	G
176	0.71	0.22	0.39	98.69	T
177	5.35	0.67	0.06	93.92	T
178	94.3	5.15	0.08	0.47	A
179	6.2	93.51	0.08	0.2	C
180	98.62	0.37	0.19	0.81	A
181	94.03	0.05	0.73	5.19	A
182	1.11	0.07	5.19	93.64	T
183	5.2	0.25	93.49	1.07	G
184	93.77	0.66	0.45	5.12	A

185	1.13	5.13	0.22	93.52	T
186	5.36	93.54	0.67	0.43	C
187	94.1	0.51	5.17	0.22	A
188	5.48	0.7	93.58	0.24	G
189	93.6	5.25	0.4	0.74	A
190	0.58	94.12	0.1	5.2	C
191	0.73	5.5	0.17	93.6	T
192	5.24	93.62	0.67	0.47	C
193	93.73	1.01	5.17	0.09	A
194	1.1	5.13	93.56	0.2	G
195	5.3	93.47	1.04	0.18	C
196	94.25	0.41	5.15	0.19	A
197	5.58	0.09	93.48	0.85	G
198	93.59	0.22	0.37	5.82	A
199	0.55	0.69	0.09	98.67	T
200	0.84	5.15	0.05	93.96	T
201	5.94	93.48	0.08	0.5	C
202	99.23	0.49	0.07	0.22	A
203	99.14	0.06	0.16	0.64	A
204	94.05	0.09	0.61	5.26	A
205	1.14	0.12	5.11	93.63	T
206	5.79	0.06	93.44	0.71	G
207	98.82	0.06	0.42	0.7	A
208	94.7	0.11	0.02	5.16	A
209	6.29	0.15	0.02	93.54	T
210	99.49	0.05	0.04	0.42	A
211	99.82	0.08	0.08	0.02	A
212	99.77	0.14	0.07	0.02	A
213	99.18	0.67	0.1	0.05	A
214	94.61	5.19	0.15	0.04	a
215	5.71	93.57	0.68	0.04	C
216	94.39	0.42	5.16	0.02	A
217	6.27	0.02	93.63	0.08	G
218	99.32	0.04	0.57	0.07	A
219	98.87	0.19	0.88	0.05	A
220	93.93	0.13	5.88	0.07	a
221	0.64	0.04	99.29	0.03	G

222	0.68	0.09	99.15	0.08	G
223	5.25	0.13	94.54	0.08	g
224	93.69	0.66	5.58	0.07	A
225	1.02	5.19	93.71	0.09	G
226	5.26	93.56	1.09	0.09	C
227	93.74	0.4	5.79	0.07	A
228	1.19	0.02	98.7	0.09	G
229	5.98	0.02	93.96	0.04	G
230	99.42	0.03	0.44	0.11	A
231	99.8	0.09	0.07	0.03	A
232	99.66	0.13	0.08	0.13	A
233	99.16	0.68	0.07	0.09	A
234	94.55	5.14	0.18	0.13	A
235	5.55	93.56	0.8	0.08	C
236	93.7	0.48	5.75	0.07	A
237	1.04	0.1	98.67	0.19	G
238	5.27	0.14	93.94	0.65	G
239	93.66	0.68	0.48	5.19	A
240	0.99	5.25	0.08	93.68	T
241	5.22	93.72	0.03	1.03	C
242	93.63	1.04	0.07	5.27	A
243	0.41	5.15	0.21	94.23	T
244	0.14	93.6	0.76	5.5	C
245	0.24	0.38	5.82	93.56	T
246	0.87	0.01	98.72	0.4	G
247	5.91	0.01	93.96	0.12	G
248	99.33	0.02	0.45	0.19	A
249	99.07	0.06	0.2	0.67	A
250	93.97	0.11	0.63	5.29	A
251	1.05	0.2	5.42	93.33	T
252	5.56	0.73	93.33	0.39	G
253	94	5.52	0.45	0.03	A
254	6.59	93.27	0.04	0.1	C
255	98.62	1.18	0.1	0.1	A
256	93.47	6.36	0.13	0.05	A
257	1.17	98.52	0.26	0.05	C
258	5.98	93.09	0.87	0.06	C

259	93.23	0.52	6.16	0.09	A
260	6.5	0.22	93.26	0.02	G
261	92.44	0.9	6.62	0.04	A
262	0.7	6.95	92.3	0.05	G
263	0.79	98.6	0.53	0.08	C
264	6.5	93.39	0.02	0.09	C
265	99.35	0.53	0.1	0.03	A
**sequence**					**length**
AGTTCCAGTATCAACCTGGCTATTCTAACTGTATGAAGAGGAAGTAGATTTATTGTTATAGTTACCTGGAGTGATACACCAAATATTCTAGTTGATGTTGGAACAAGTTGAACTATGCCAATATAGCTAAAATAACTAAAATCTACTCCAGAATTCTGAAGGTGTAACTGGGAGGTTACAATGATCAGACTCAGCAGATTCAAATGAATAAAAaCAGAAaGGgAGCAGGAAAAACAGGATCATCTGGAAATGACAACCAGAGCCA					265
**total reads**	**perfect reads**	**off-target editing ratio**			
2688523	2679278	0.34%			
**Ni OT-3**					
location	A	C	G	T	ref base
1	97.98	0.22	1.69	0.11	A
2	0.2	1.97	97.55	0.27	G
3	0.04	97.35	0.3	2.31	C
4	0.11	0.24	2.46	97.18	T
5	0.44	0.23	96.44	2.88	G
6	3.04	0.6	0.16	96.19	T
7	95.75	3.83	0.11	0.32	A
8	0.17	99.14	0.09	0.61	C
9	0.04	95.39	0.1	4.48	C
10	0.11	5	0.16	94.74	T
11	0.09	94.27	0.34	5.3	C
12	0.23	5.74	1.05	92.97	T
13	1	92.75	6.07	0.18	C
14	6.06	0.37	93.45	0.12	G
15	92.78	1	6.07	0.15	A
16	1.16	6.03	92.43	0.38	G
17	6.04	92.57	0.16	1.23	C
18	92.58	0.18	0.06	7.19	A
19	0.39	0.07	0.03	99.51	T
20	0.91	0.13	0.03	98.92	T
21	6.09	0.09	0.01	93.81	T

22	92.45	0.22	0.06	7.26	A
23	0.29	0.12	0.06	99.52	T
24	0.05	0.44	0.06	99.45	T
25	0.21	1.25	0.04	98.5	T
26	0.14	7.03	0.09	92.75	T
27	0.09	98.33	0.39	1.19	C
28	0.19	92.63	0.9	6.29	C
29	0.36	0.2	6.04	93.39	T
30	0.98	0.08	92.34	6.6	G
31	6.04	0.13	0.23	93.6	T
32	92.41	0.29	0.07	7.22	A
33	0.32	0.45	0.05	99.18	T
34	0.29	1.16	0.07	98.48	T
35	1.13	6.06	0.06	92.75	T
36	6.11	92.74	0.08	1.08	C
37	92.66	1.23	0.08	6.04	A
38	1.44	5.99	0.14	92.42	T
39	6.97	92.49	0.32	0.22	C
40	98.26	0.37	1.2	0.17	A
41	92.47	0.01	6.91	0.6	A
42	0.25	0.03	98.36	1.36	G
43	0.19	0.16	92.44	7.21	G
44	0.23	0.17	0.19	99.41	T
45	0.16	0.21	0.05	99.57	T
46	0.48	0.08	0.08	99.35	T
47	1.23	0.06	0.25	98.46	T
48	7.18	0.11	0.31	92.4	T
49	99.18	0.06	0.47	0.28	A
50	98.52	0.05	1.14	0.3	A
51	92.61	0.13	6.32	0.94	A
52	0.33	0.35	93.28	6.03	G
53	0.28	1.05	6.3	92.37	T
54	0.99	6.04	92.63	0.34	G
55	6.28	92.36	1.31	0.05	C
56	92.6	0.34	6.94	0.12	A
57	1.34	0.16	98.29	0.21	G
58	7.18	0.06	92.48	0.28	G

59	98.38	0.17	0.46	0.99	A
60	92.59	0.16	1.08	6.17	A
61	1.16	0.06	6.08	92.7	T
62	6.13	0.09	92.44	1.34	G
63	92.54	0.11	0.28	7.07	A
64	1.23	0.4	0.06	98.31	T
65	6.28	0.98	0.11	92.63	T
66	93.33	6.21	0.25	0.22	A
67	6.48	92.46	0.91	0.15	C
68	93.3	0.22	6.06	0.42	A
69	6.47	0.07	92.34	1.12	G
70	93.47	0.16	0.26	6.11	A
71	7.41	0.09	0.19	92.31	T
72	99.29	0.19	0.28	0.24	A
73	98.7	0.18	1.09	0.03	A
74	93.51	0.07	6.24	0.19	A
75	7.07	0.31	92.49	0.13	G
76	98.35	0.43	1.04	0.18	A
77	92.53	1.13	6.05	0.29	A
78	0.52	6.07	92.31	1.09	G
79	1.13	92.39	0.25	6.23	C
80	6.93	0.54	0.1	92.43	T
81	97.98	1.67	0.13	0.22	A
82	92.63	7.04	0.1	0.24	A
83	1.19	98.26	0.13	0.42	C
84	6.18	92.56	0.29	0.96	C
85	92.49	0.16	1.12	6.24	A
86	0.51	0.2	6.99	92.29	T
87	1.26	0.18	98.27	0.29	G
88	7.26	0.23	92.36	0.16	G
89	99.55	0.08	0.19	0.18	A
90	99.33	0.25	0.17	0.25	A
91	98.75	0.08	0.1	1.07	A
92	93.55	0.31	0.06	6.07	A
93	6.7	0.92	0.01	92.37	T
94	93.4	6.19	0.05	0.37	A
95	6.7	92.23	0.11	0.95	C

96	93.53	0.15	0.18	6.14	A
97	7.57	0.02	0.12	92.29	T
98	99.59	0.06	0.09	0.27	A
99	99.55	0.03	0.07	0.35	A
100	98.62	0.16	0.07	1.15	A
101	92.8	0.08	0.04	7.08	A
102	1.42	0.04	0.05	98.49	T
103	7.32	0.22	0.06	92.41	T
104	99.62	0.06	0.1	0.22	A
105	99.57	0.11	0.21	0.11	A
106	99.58	0.13	0.02	0.27	A
107	98.55	0.28	0.05	1.12	A
108	92.76	1.06	0.07	6.11	A
109	1.29	6.19	0.04	92.47	T
110	6.5	92.29	0.12	1.08	C
111	93.3	0.38	0.06	6.26	A
112	6.37	1.08	0.1	92.45	T
113	92.54	6.07	0.25	1.15	A
114	0.51	92.36	1.07	6.07	C
115	1.06	0.53	6.08	92.33	T
116	6.27	1.04	92.42	0.27	G
117	93.16	6.06	0.49	0.28	A
118	6.19	92.46	1.06	0.29	C
119	92.31	0.18	6.35	1.17	A
120	0.19	0.02	93.55	6.24	G
121	0.13	0.08	7.16	92.63	T
122	0.09	0.07	98.5	1.34	G
123	0.3	0.05	93.34	6.3	G
124	1.05	0.03	6.28	92.64	T
125	6.19	0.06	92.66	1.09	G
126	92.4	0.17	1.29	6.14	A
127	0.4	0.07	6.94	92.59	T
128	0.2	0.12	98.39	1.28	G
129	0.47	0.14	92.43	6.96	G
130	1.03	0.3	0.32	98.35	T
131	6.39	1.13	0.1	92.38	T
132	93.5	6.25	0.09	0.15	A

133	6.53	93.27	0.16	0.04	C
134	93.62	6.27	0.08	0.03	A
135	7.15	92.62	0.16	0.06	C
136	98.53	1.32	0.07	0.08	A
137	93.55	6.23	0.14	0.09	A
138	6.53	93.18	0.24	0.05	C
139	93.31	6.34	0.22	0.13	A
140	6.37	92.4	0.97	0.26	C
141	92.67	0.32	6.08	0.93	A
142	1.4	0.01	92.31	6.28	G
143	7.19	0.04	0.24	92.53	T
144	99.21	0.03	0.3	0.46	A
145	98.6	0.07	0.36	0.97	A
146	92.68	0.09	0.97	6.26	A
147	1.36	0.07	6.17	92.4	T
148	7.15	0.12	92.18	0.56	G
149	99.14	0.16	0.24	0.45	A
150	98.34	0.38	0.29	0.99	A
151	92.4	1.37	0.14	6.09	A
152	1.13	6.09	0.42	92.36	T
153	6.11	92.46	1.06	0.36	C
154	92.34	0.35	6.27	1.04	A
155	0.22	0.31	93.28	6.19	G
156	0.19	1.16	6.26	92.4	T
157	0.3	6.99	92.37	0.34	G
158	1.09	98.54	0.24	0.13	C
159	6.16	93.42	0.12	0.31	C
160	92.51	6.26	0.24	0.99	A
161	0.51	92.43	0.94	6.12	C
162	1.14	0.28	6.07	92.51	T
163	7.09	0.22	92.35	0.35	G
164	98.49	0.95	0.24	0.31	A
165	92.73	6.1	0.09	1.08	A
166	1.2	92.38	0.25	6.16	C
167	6.35	0.19	0.99	92.47	T
168	93.39	0.13	6.06	0.42	A
169	6.4	0.08	92.27	1.25	G

170	93.32	0.28	0.25	6.15	A
171	6.24	0.95	0.16	92.65	T
172	92.48	6.04	0.06	1.42	A
173	0.54	92.37	0.14	6.95	C
174	1.34	0.2	0.18	98.28	T
175	7.19	0.16	0.16	92.49	T
176	99.44	0.1	0.08	0.39	A
177	99.29	0.18	0.14	0.39	A
178	98.12	0.57	0.29	1.03	A
179	92	0.75	1.18	6.07	A
180	0.28	0.28	7.07	92.37	T
181	0.2	1.17	98.35	0.27	G
182	0.41	6.97	92.43	0.19	G
183	1.3	98.38	0.18	0.15	C
184	7.05	92.47	0.09	0.39	C
185	98.36	0.24	0.11	1.29	A
186	91.7	1	0.11	7.19	A
187	0.32	0.03	1.3	98.36	T
188	0.29	0.03	0.47	99.21	T
189	0.96	0.09	0.38	98.58	T
190	6.11	0.04	0.28	93.58	T
191	92.58	0.01	0.98	6.44	A
192	0.5	0.04	6.06	93.41	T
193	1.23	0.01	92.42	6.34	G
194	7.17	0.05	0.2	92.58	T
195	99.37	0.04	0.12	0.47	A
196	98.63	0.07	0.28	1.02	A
197	92.58	0.16	0.93	6.33	A
198	0.4	0.15	6.06	93.39	T
199	1.01	0.24	92.44	6.31	G
200	6.26	0.96	0.18	92.6	T
201	93.35	6.09	0.02	0.54	A
202	6.25	92.45	0.05	1.25	C
203	92.49	0.22	0.04	7.25	A
204	0.31	0.14	0.01	99.54	T
205	0.27	0.18	0.02	99.52	T
206	0.94	0.34	0.19	98.54	T

207	6.16	1.2	0.21	92.43	T
208	92.6	7.16	0.06	0.19	A
209	0.48	99.39	0.05	0.09	C
210	1.25	98.63	0.01	0.11	C
211	7.08	92.62	0.01	0.29	C
212	98.61	0.22	0.04	1.14	A
213	92.74	0.21	0.02	7.03	A
214	1.37	0.04	0.08	98.51	T
215	7.21	0.05	0.15	92.59	T
216	99.45	0.13	0.07	0.35	A
217	98.71	0.33	0.05	0.91	A
218	93.59	0.27	0.07	6.07	A
219	7.38	0.05	0.09	92.48	T
220	99.64	0.08	0.12	0.16	A
221	99.51	0.23	0.24	0.02	A
222	98.6	0.43	0.94	0.03	A
223	92.68	1.17	6.12	0.03	A
224	0.27	7.17	92.48	0.08	G
225	0.32	99.44	0.19	0.05	C
226	1.12	98.71	0.06	0.11	c
227	7	92.63	0.16	0.21	c
228	98.5	0.25	0.32	0.92	A
229	92.5	0.21	1.16	6.12	A
230	0.23	0.28	7.14	92.35	T
231	0.23	0.98	98.62	0.18	G
232	0.92	6.15	92.87	0.06	G
233	6.09	92.68	1.13	0.1	c
234	92.47	1.1	6.19	0.24	A
235	0.15	6.17	92.74	0.93	G
236	0.04	92.6	1.26	6.11	C
237	0.01	0.43	7.03	92.53	T
238	0.02	0.97	98.6	0.42	G
239	0.05	6.27	92.71	0.98	G
240	0.04	93.37	0.31	6.29	C
241	0.08	6.25	0.32	93.35	T
242	0.12	92.49	1.18	6.21	C
243	0.08	0.2	7.25	92.48	T

244	0.22	0.08	99.49	0.2	G
245	0.93	0.13	98.88	0.05	G
246	6.18	0.24	93.55	0.03	G
247	92.68	0.95	6.32	0.04	A
248	1.07	6.18	92.71	0.04	G
249	6.11	92.71	1.1	0.09	C
250	92.47	1.34	6.11	0.08	A
251	0.15	7.19	92.46	0.19	G
252	0.03	99.41	0.23	0.33	C
253	0.08	98.59	0.13	1.2	C
254	0.03	92.16	0.36	7.46	C
255	0.04	0.32	1.33	98.31	T
256	0.04	0.02	8.43	91.5	T
257	0.02	0.06	98.3	1.62	G
258	0.03	0.02	91.29	8.65	G
259	0.07	0.01	9.18	90.73	T
260	0.12	0	88.83	11.04	G
261	0.4	0.01	2.11	97.48	T
262	1.76	0.02	10.65	87.57	T
263	10.51	0.02	89.08	0.38	G
264	88.36	0.02	11.54	0.07	A
265	0.46	0	99.44	0.09	G
**sequence**					**length**
AGCTGTACCTCTCGAGCATTTATTTTCCTGTATTTCATCAAGGTTTTTAAAGTGCAGGAATGATTACAGATAAAGAAGCTAACCATGGAAAATACATAAAATTAAAAATCATACTGACAGTGGTGATGGTTACACAACACAGTAAATGAAATCAGTGCCACTGAACTAGATACTTAAAATGGCCAATTTTATGTAAATGTACATTTTACCCAATTAAATAAAAGCccAATGGcAGCTGGCTCTGGGAGCAGCCCTTGGTGTTGAG					265
**total reads**	**perfect reads**	**off-target editing ratio**			
2801912	2794490	0.26%			
